# Biometric Identification Systems with Noisy Enrollment for Gaussian Sources and Channels †

**DOI:** 10.3390/e23081049

**Published:** 2021-08-15

**Authors:** Vamoua Yachongka, Hideki Yagi, Yasutada Oohama

**Affiliations:** 1 Advanced Wireless & Communication Research Center (AWCC), The University of Electro-Communications, 1-5-1 Chofugaoka, Tokyo 182-8585, Japan; 2 Department of Computer and Network Engineering, The University of Electro-Communications, 1-5-1 Chofugaoka, Tokyo 182-8585, Japan; h.yagi@uec.ac.jp (H.Y.); oohama@uec.ac.jp (Y.O.)

**Keywords:** biometric identification system, noisy enrollment, privacy-leakage, entropy power inequality

## Abstract

In the present paper, we investigate the fundamental trade-off of identification, secret-key, storage, and privacy-leakage rates in biometric identification systems for remote or hidden Gaussian sources. We use a technique of converting the system to one where the data flow is in one-way direction to derive the capacity region of these rates. Also, we provide numerical calculations of three different examples for the system. The numerical results imply that it seems hard to achieve both high secret-key and small privacy-leakage rates simultaneously.

## 1. Introduction

Biometric identification indicates an automated process of recognizing an individual by matching the individual’s biological data (bio-data) with the digital files stored in the system database [1]. Some unique bio-data that can be used for biometric identification include fingerprint, iris, face, voice, palm, and so on [2]. Compared to the traditional method such as password or smart card based identification, it provides higher convenience and security. However, the critical drawback for biometric identification is that the usable sources are limited [3], for instance, human has only two eyes and if their information are leaked, there is no alternative option to replace, and therefore it is important to protect users’ privacy. Furthermore, the size of the storage should be minimized to reduce the memory space of the database [4], especially when the number of users becomes large.

From an information theoretic point of view, there are two major settings of the studies related to biometric identification systems (BISs), namely, the BIS with exponentially many users and the system with one user. The difference of these systems is that in the former setting, we are interested in finding the maximum number of users who are reliably identifiable, i.e., the maximum achievable identification rate at which the error probability of the BIS vanishes (the identification capacity). However, in the latter setting, the estimation of the user need not be considered since there exists only one user and it becomes redundant. For discrete memoryless sources (DMSs), the fundamental performance of the BIS was widely analyzed for both scenarios.

The BIS with multiple users was initially treated as a mathematical model in the seminal work [5], and the identification capacity of the BIS was clarified. In the model, it is assumed that every biometric identifier is enrolled via a noisy channel, and this type of model is known as a remote or hidden source model. The term the remote sources were used in [6,7], and hidden source model (HSM) is from [8]. In this paper, we use HSM as in [8] to represent the BIS with noisy enrollment. The encoding process was introduced in [9] to reduce the size of the storage. This work was extended to incorporate noisy reconstruction in [10]. The BIS with estimating both user’s index and secret key for two classical models, namely, *generated-* and *chosen-secret* BIS models, was investigated in [11]. In this literature, a clear explanation of the difference of these models is given. Later, adopting the concept of the wiretap channel to assume that the adversary has side information of the identified user’s bio-data sequence for the generated-secret BIS model was analyzed in [12]. Recently, a storage constraint and an HSM added to the model of [11] were studied in [13,14]. By using an additional private key, user’s privacy-leakage can be made negligible [15,16]. Another scenario, that is, the BIS with one user, was extensively examined in [8,17,18,19,20,21,22]. More precisely, in [17,18], the relation of secret-key and privacy-leakage rates was analyzed. The optimal secret-key rate under privacy and storage constraints was characterized in [8,19] for non-vanishing and vanishing secrecy-leakage rate, respectively. It is worthwhile noting that in [8], a successful attempt for characterizing the capacity region of the BIS with one user for HSM was first made. The works of [8] was extended to constrain the action cost for the decoder in [20], and to consider two-enrollment systems for the same hidden source, where the encoders do not trust each other [21]. Moreover, in [22], the secret-key capacity of a multi-enrollment system, in which the decoder is required to estimate all secret keys generated in the earlier enrollments, was formulated.

Compared to the analyses of the BIS for DMSs, the results given under Gaussian sources are still few. For example, the optimal trade-off between secret-key and privacy-leakage rates was characterized in [23] and in order to speed up search complexity, hierarchical identification was taken into account in [24]. A common assumption in [23,24] is that the enrollment channel is noiseless, known as a visible source model (VSM). However, in real-life application, the signal of bio-data is basically represented with continuous values, and most communication links can be modeled as Gaussian channels [23]. What is more, the HSM is considered to be more realistic, e.g., captured picture of a finger via a scanner, and when the BIS is switched from the VSM to the HSM, the evaluation becomes more challenging [8] because many techniques used for deriving the results of the VSM are not directly applicable. These facts motivate us to extend the models in [13] to Gaussian sources and channels. Note that from the technical perspectives, this extension is not trivial since the technique for establishing Theorems 1 and 2 in [13] massively depends on the property that the alphabet sizes are finite, but unfortunately it cannot be applied to continuous sources. The technique used in this paper will be explained in Section 5 in details. Therefore, the extension is of both theoretical and practical interest. Although it is well-known that the bio-data is real-valued, as mentioned in [23], the validity of Gaussian assumption is not discussed in this paper and we leave this for further research. Here, we are interested in specifying the optimal trade-off of the BIS.

In this study, our goal is to find the optimal trade-off of identification and secret-key rates in the BIS under privacy and storage constraints. We demonstrate that an idea of converting the system to another one, where the data flow of each user is in the same direction, enables us to characterize the capacity region. More specifically, in establishing the outer bound of the region, the converted system allows us to use the entropy power inequality (EPI) [25] doubly in two opposite directions, and also its property facilitates the derivation of the inner bound. In [8], Mrs. Gerber’s lemma was applied twice, too, to simplify the rate region of the HSM for binary sources and symmetric channels without converting the BIS. That was possible due to the uniformity of the source, and the backward channel of the enrollment channel is also the binary symmetric channel with the same crossover probability. However, this claim is no longer true in the Gaussian case, so it is necessary to formulate the general behavior of the backward channel. We also provide numerical calculations of three different examples. As a consequence, we may conclude that it is difficult to achieve high secret-key and small privacy-leakage rates at the same time. To achieve a small privacy-leakage rate, the secret-key rate must be sacrificed. Furthermore, as a by-product of our result, the capacity regions of the BIS analyzed in [8] for Gaussian sources and channels are obtained, and as special cases, it can be checked that this characterization reduces to the results given in [5,23].

The rest of this paper is organized as follows. In Section 2, we define the notation used in this paper, and describe our system model and the converted system. In Section 3, the formal definitions and main results are discussed in detail. We continue investigating the basic properties of the capacity regions, and provide there different examples in Section 4. The overviews of the proof of our main results are given in Section 5. The full proof is available in Appendix A and Appendix B. Finally, some concluding remark and future work are mentioned in Section 6.

## 2. System Model and Converted System

### 2.1. Notation and System Model

Upper-case *A* and lower-case *a* denote random variable (RV) and its realization, respectively. $A^{n}=\left(A_{1},\cdots ,A_{n}\right)$ represents a string of RVs and subscripts represent the position of an RV in the string. $f_{A}$ denotes the probability density function (pdf) of RV *A*. For integers *k* and *t* such that k<t, [k:t] denotes the set {k,k+1,⋯,t}. logx stands for the natural logarithm of x>0.

**Figure 1:**
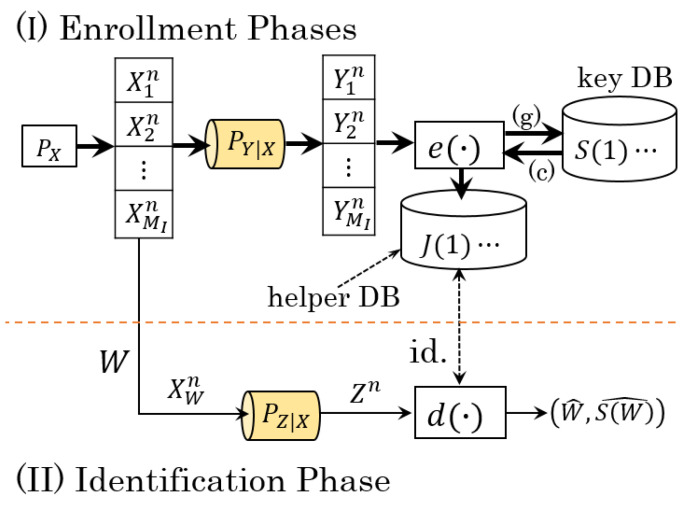
The generated- and chosen-secret BIS models.

The generated-secret BIS model and chosen-secret BIS model considered in this study are depicted in Figure 1. Arrows (g) and (c) indicate the directions of the secret key of the former and latter models. Let $I=[1:M_{I}]$, $S=[1:M_{S}]$, and $J=[1:M_{J}]$ be the sets of user’s indices, secret keys, and helper data, respectively, where $M_{I}$, $M_{S}$, and $M_{J}$ denote the numbers of users, secret keys, and helper data, respectively. These sets are assumed to be finite. $X_{i}^{n}$,$Y_{i}^{n}$, and $Z^{n}$ denote the bio-data sequence of user *i* generated from source $P_{X}$, the output of $X_{i}^{n}$ via the enrollment channel $P_{Y|X}$, and the output of $X_{i}^{n}$ via the identification channel $P_{Z|X}$, respectively. For i∈I and k∈[1:n], we assume $X_{ik}∼N\left(0,1\right)$, where N(0,1) is a Gaussian RV with mean zero and variance one. Note that an RV with unit variance can be obtained by applying a scaling technique. $P_{Y|X}$ and $P_{Z|X}$ are additive Gaussian noise channels modeled as follows:

(1) $$\begin{array}{c} Y_{ik}=\rho_{1}X_{ik}+N_{1},\;\;\;\;\;Z_{k}=\rho_{2}X_{ik}+N_{2},\;\;\;\;\;\;\;\;\left(k\in [1:n]\right). \end{array}$$

where $|\rho_{1}|<1$, $|\rho_{2}|<1$ are the Pearson’s correlation coefficients, and $N_{1}∼N\left(0,1-\rho_{1}^{2}\right)$ and $N_{2}∼N\left(0,1-\rho_{2}^{2}\right)$ are Gaussian RVs, independent of each other and bio-data sequences. From (1), $Y_{ik}$ and $Z_{k}$ are also Gaussian with zero mean and unit variance, and the Markov chain Y−X−Z holds. Then, the pdf corresponding to the tuple $\left(X_{i}^{n},Y_{i}^{n},Z^{n}\right)$ is given by

(2) $$\begin{array}{c} f_{X_{i}^{n}Y_{i}^{n}Z^{n}}\left(x_{i}^{n},y_{i}^{n},z^{n}\right)=\prod_{k=1}^{n}f_{XYZ}\left(x_{ik},y_{ik},z_{k}\right), \end{array}$$

where for x,y,z∈R,

(3) $$\begin{array}{cc} \;f_{XYZ}\left(x,y,z\right) & =f_{X}\left(x\right)\cdot f_{Y|X}\left(y|x\right)\cdot f_{Z|X}\left(z|x\right), \end{array}$$

(4) $$\begin{array}{cc} \; & =\frac{1}{\sqrt{{\left(2\pi \right)}^{3}\left(1-\rho_{1}^{2}\right)\left(1-\rho_{2}^{2}\right)}}\exp\left(-\left(\frac{x^{2}}{2}+\frac{{\left(y-\rho_{1}x\right)}^{2}}{2(1-\rho_{1}^{2})}+\frac{{\left(z-\rho_{2}x\right)}^{2}}{2(1-\rho_{2}^{2})}\right)\right). \end{array}$$

In the generated-secret BIS model, upon observing $Y_{i}^{n}$, the encoder e(·) generates secret key S(i)∈S and helper data J(i)∈J as $\left(S\left(i\right),J\left(i\right)\right)=e\left(Y_{i}^{n}\right)$. Then, J(i) is stored at position *i* in the public database (helper DB) and S(i) is saved in the key DB, which is installed in a secure location. Let *W* and $\hat{W}$ denote the index of the identified user and its estimated value, respectively. Seeing $Z^{n}$, the decoder d(·) estimates $\left(\hat{W},\hat{S(W)}\right)$ from $Z^{n}$ and all helper data in DB $J\equiv \{J\left(1\right),\cdots ,J\left(M_{I}\right)\}$, i.e., $\left(\hat{W},\hat{S(W)}\right)=d\left(Z^{n},J\right)$.

In the chosen-secret BIS model, the secret key S(i) is chosen uniformly from S, i.e.,

(5) $$\begin{array}{c} P_{S(i)}\left(s\right)=1/M_{S}\;\;\;\;\;\;\;\;\;\left(s\in S\right), \end{array}$$

and independent of other RVs. The encoder forms the helper data as $J\left(i\right)=e(Y_{i}^{n},S\left(i\right))$ for every individual. The decoder d(·) owns the same functionality as in the generated-secret BIS model.

### 2.2. Converted System

**Figure 2:**
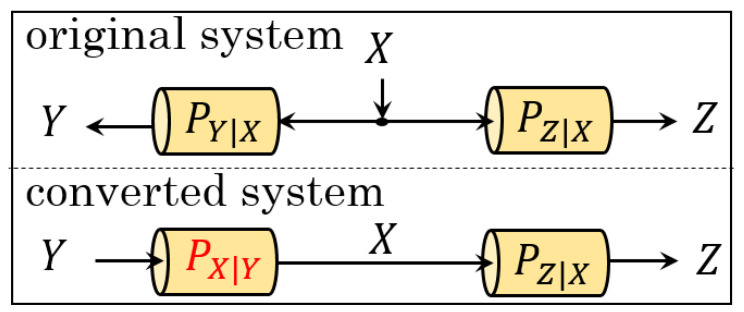
The original (**top**) and converted (**bottom**) systems.

The original system, having *X* as input source and Y,Z as outputs, is in the top figure in Figure 2. There are two main obstacles toward characterizing the capacity regions directly from this system. (I) In establishing the converse proof, an upper bound regarding RV *Y* for a fixed condition of RV *X* is needed, but it is laborious to pursue the desired bound since applying EPI to the first relation in (1) produces only a lower bound. (II) It seems difficult to prove the achievability part by generating the codebook via a test channel due to the input *X*. To overcome these bottlenecks, we use an idea of converting the original system to a new one in which the data flow of each user is one-way from *Y* to *Z* without losing its general properties. The image of this idea is shown in the bottom figure of Figure 2, where *Y* becomes input virtually. To achieve this objective, knowing the statistics of the backward channel $P_{X|Y}$, namely, how *X* correlates to the virtual input *Y*, is crucial and we explore that in the rest of this section.

Due to the Markov chain Y−X−Z, Equation (3) can also be expanded in the following form.

(6) $$\begin{array}{c} f_{XYZ}\left(x,y,z\right)=f_{Y}\left(y\right)\cdot f_{X|Y}\left(x|y\right)\cdot f_{Z|X}\left(z|x\right). \end{array}$$

Observe that

(7) $$\begin{array}{cc} \frac{x^{2}}{2}+\frac{{\left(y-\rho_{1}x\right)}^{2}}{2(1-\rho_{1}^{2})} & =\frac{x^{2}}{2}+\frac{y^{2}}{2(1-\rho_{1}^{2})}-\frac{\rho_{1}xy}{1-\rho_{1}^{2}}+\frac{{\left(\rho_{1}x\right)}^{2}}{2(1-\rho_{1}^{2})}=\frac{y^{2}}{2}+\frac{{\left(x-\rho_{1}y\right)}^{2}}{2(1-\rho_{1}^{2})}. \end{array}$$

Without loss of generality, the exponential part in (4) can be rearranged as

(8) $$\begin{array}{c} -\left(\frac{y^{2}}{2}+\frac{{\left(x-\rho_{1}y\right)}^{2}}{2(1-\rho_{1}^{2})}+\frac{{\left(z-\rho_{2}x\right)}^{2}}{2(1-\rho_{2}^{2})}\right). \end{array}$$

From (6) and (8), we may conclude that the following relations hold with some RV $N_{1}^{\prime }∼N\left(0,1-\rho_{1}^{2}\right)$.

(9) $$\begin{array}{cc} X_{ik} & =\rho_{1}Y_{ik}+N_{1}^{\prime }, \end{array}$$

(10) $$\begin{array}{cc} Z_{k} & =\rho_{2}X_{ik}+N_{2}=\rho_{1}\rho_{2}Y_{ik}+\rho_{2}N_{1}^{\prime }+N_{2}. \end{array}$$

Equations (9) and (10) describe the outputs of the backward channel $P_{X|Y}$ and the combined channel $P_{Z|Y}$ of the virtual system. Actually, these relations can also be observed directly from the covariance matrix of RVs (X,Y,Z). However, we derive them based on the joint pdf for general readers’ purpose. Moreover, this transformation is useful for the analysis of a non-standard source. The above relations play key roles for solving the problem of the HSM, and we use them in many steps during the analysis in this paper. In [23,24], the concept of this transformation is not seen because the enrollment channel is noiseless due to the assumption of VSM as mentioned before.

**Remark** **1.** *In the case where there is no operation of scaling, Equations (9) and (10) are settled as follows. Suppose that $X_{ik}∼N\left(0,\sigma_{x}^{2}\right)$ with $\sigma_{x}^{2}<\infty$, $Y_{ik}=X_{ik}+D_{1}$, and $Z_{k}=X_{ik}+D_{2}$, where $D_{1}∼N\left(0,\sigma_{1}^{2}\right)$ and $D_{2}∼N\left(0,\sigma_{2}^{2}\right)$ are Gaussian RVs, and independent of each other and other RVs. By applying the similar arguments around (6)–(8), we obtain that*

(11) $$\begin{array}{c} X_{ik}=\frac{\sigma_{x}^{2}}{\sigma_{x}^{2}+\sigma_{1}^{2}}Y_{ik}+D_{1}^{\prime },\;\;\;\;\;Z_{k}=X_{ik}+N_{2}^{\prime }=\frac{\sigma_{x}^{2}}{\sigma_{x}^{2}+\sigma_{1}^{2}}Y_{ik}+D_{1}^{\prime }+D_{2}, \end{array}$$

*where $D_{1}^{\prime }∼N\left(0,\frac{\sigma_{x}^{2}\sigma_{1}^{2}}{\sigma_{x}^{2}+\sigma_{1}^{2}}\right)$ is Gaussian and independent of other RVs. The capacity regions of the models considered in this paper can also be characterized via (11), and the results for this case will be mentioned in Remark 3. However, equation developments need more space and do not look so neat. Herein, we pursue our results based on the method that RVs X, Y, and Z are standardized.*

Now from (9) and (10), it is not difficult to verify that

(12) $$\begin{array}{c} I\left(X;Y\right)=\frac{1}{2}\log\left(\frac{1}{1-\rho_{1}^{2}}\right),\;\;\;\;\;I\left(Z;Y\right)=\frac{1}{2}\log\left(\frac{1}{1-\rho_{1}^{2}\rho_{2}^{2}}\right), \end{array}$$

where the right equation in (12) is attained because the variance of the noise term $\rho_{2}N_{1}^{\prime }+N_{2}$ in (10) is equal to $1-\rho_{1}^{2}\rho_{2}^{2}$.

## 3. Problem Formulation and Main Results

The achievability definition for the generated-secret BIS model is given below.

**Definition** **1.** *A tuple of identification, secret-key, public storage, and privacy-leakage rates $\left(R_{I},R_{S},R_{J},R_{L}\right)$ is said to be achievable for the generated-secret BIS model under a Gaussian source if for any δ>0 and large enough n there exist pairs of encoders and decoders satisfying*

(13) $$\begin{array}{cc} \max_{i\in I}\mathrm{Pr}\{(\hat{W},\hat{S(W))}\neq \left(W,S\left(W\right)\right) & |W=i\}\leq \delta ,\;\;\;\;\;\;\;\;\;\;\;\;\left(\mathrm{error}\;\;\mathrm{probability}\right) \end{array}$$

(14) $$\begin{array}{cc} \frac{1}{n}\log M_{I} & \geq R_{I}-\delta ,\;\;\;\;\;\;\;\;\;\;\;\;\;\;\;\;\;\left(\mathrm{identification}\;\;\mathrm{rate}\right) \end{array}$$

(15) $$\begin{array}{cc} \min_{i\in I}\frac{1}{n}H\left(S\left(i\right)\right) & \geq R_{S}-\delta ,\;\;\;\;\;\;\;\;\;\;\;\;\;\;\;\;\;\left(\mathrm{secret}-\mathrm{key}\;\;\mathrm{rate}\right) \end{array}$$

(16) $$\begin{array}{cc} \frac{1}{n}\log M_{J} & \leq R_{J}+\delta ,\;\;\;\;\;\;\;\;\;\;\;\;\;\;\;\;\;\left(\mathrm{public}\;\;\mathrm{storage}\;\;\mathrm{rate}\right) \end{array}$$

(17) $$\begin{array}{cc} \max_{i\in I}\frac{1}{n}I\left(S\left(i\right);J\left(i\right)\right) & \leq \delta ,\;\;\;\;\;\;\;\;\;\;\;\;\;\;\;\;\;\;\;\;\;\;\;\;\;\;\left(\mathrm{secrecy}-\mathrm{leakage}\;\;\mathrm{rate}\right) \end{array}$$

(18) $$\begin{array}{cc} \max_{i\in I}\frac{1}{n}I\left(X_{i}^{n};J\left(i\right)\right) & \leq R_{L}+\delta .\;\;\;\;\;\;\;\;\;\;\;\;\;\;\;\;\;\left(\mathrm{privacy}-\mathrm{leakage}\;\;\mathrm{rate}\right) \end{array}$$

*Moreover, $R_{G}$ is defined as the set of all achievable rate tuples for the generated-secret BIS model, called the capacity region.*

For the chosen-secret BIS model, the definition is provided as follows:

**Definition** **2.** *A tuple $\left(R_{I},R_{S},R_{J},R_{L}\right)$ is said to be achievable for the chosen-secret BIS model under a Gaussian source if there exist pairs of encoders and decoders that satisfy all the requirements in Definition 1 for any δ>0 and large enough n. Note that the left-hand side of (15) is expressed as $\frac{1}{n}\log M_{S}$ because the key is chosen uniformly from S (cf. (5)). In addition, $R_{C}$ is defined as the capacity region for the chosen-secret BIS model.*

**Remark** **2.** *Note that in the BIS, there are two databases, namely, databases of secret keys and helper data. The memory space of the database for storing the helper data (public database) is minimized, while that for the secret keys (secure database) should be maximized. This means only a part of the entire storage space of the BIS, which is the public database, is being compressed, and thus it is suitable to call this compression rate the public storage rate. However, we call the public storage rate just the storage rate as in* [8] *hereafter for brevity reason.*

Now we are ready to introduce our main results.

**Theorem** **1.** *The capacity regions for the generated- and chosen-secret BIS models are given by*

(19) $$\begin{array}{cc} R_{G}=\underset{0<\alpha \leq 1}{⋃}\{\left(R_{I},R_{S},R_{J},R_{L}\right):R_{I}+R_{S} & \leq \frac{1}{2}\log\left(\frac{1}{\alpha \rho_{1}^{2}\rho_{2}^{2}+1-\rho_{1}^{2}\rho_{2}^{2}}\right),\\ R_{J} & \geq \frac{1}{2}\log\left(\frac{\alpha \rho_{1}^{2}\rho_{2}^{2}+1-\rho_{1}^{2}\rho_{2}^{2}}{\alpha }\right)+R_{I},\\ R_{L} & \geq \frac{1}{2}\log\left(\frac{\alpha \rho_{1}^{2}\rho_{2}^{2}+1-\rho_{1}^{2}\rho_{2}^{2}}{\alpha \rho_{1}^{2}+1-\rho_{1}^{2}}\right)+R_{I},\\ R_{I} & \geq 0,\;\;R_{S}\geq 0\}, \end{array}$$

(20) $$\begin{array}{cc} R_{C}=\underset{0<\alpha \leq 1}{⋃}\{\left(R_{I},R_{S},R_{J},R_{L}\right):R_{I}+R_{S} & \leq \frac{1}{2}\log\left(\frac{1}{\alpha \rho_{1}^{2}\rho_{2}^{2}+1-\rho_{1}^{2}\rho_{2}^{2}}\right),\\ R_{J} & \geq \frac{1}{2}\log\left(\frac{1}{\alpha }\right),\\ R_{L} & \geq \frac{1}{2}\log\left(\frac{\alpha \rho_{1}^{2}\rho_{2}^{2}+1-\rho_{1}^{2}\rho_{2}^{2}}{\alpha \rho_{1}^{2}+1-\rho_{1}^{2}}\right)+R_{I},\\ R_{I} & \geq 0,\;\;R_{S}\geq 0\}. \end{array}$$

The proof of Theorem 1 is provided in Appendix A and Appendix B. It can be verified that the regions $R_{G}$ and $R_{C}$ are both convex, whose proofs are available in Appendix C. Unlike the approach taken in [23], based on investigating the second derivative of the rate region function, our proof makes use of the concavity of the logarithmic function. In both regions, α=0 is excluded by the reason that the point is not achievable, and this fact will be mentioned again in the converse proof of Equation (19).

**Figure 3:**
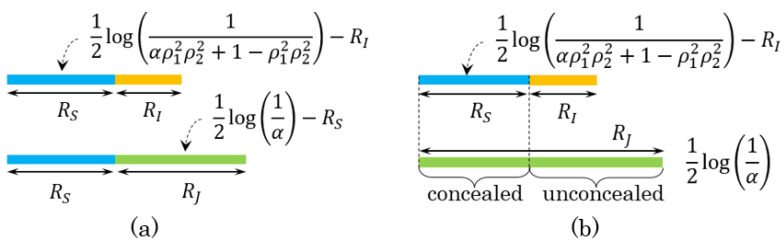
(**a**,**b**) are the explanations of the optimal values of identification, secret-key, storage, and privacy-leakage rates in the regions $R_{G}$ and $R_{C}$, respectively, for a fixed α.

For a fixed α, the optimal rate values for the regions $R_{G}$ and $R_{C}$ are shown in Figure 3. We begin with explaining Figure 3a. Suppose that $0<R_{I}<\frac{1}{2}\log\left(\frac{1}{\alpha \rho_{1}^{2}\rho_{2}^{2}+1-\rho_{1}^{2}\rho_{2}^{2}}\right)$. In the top band chart, $\frac{1}{2}\log\left(\frac{1}{\alpha \rho_{1}^{2}\rho_{2}^{2}+1-\rho_{1}^{2}\rho_{2}^{2}}\right)$ is the maximum achievable rate that user’s identities can be estimated correctly at the decoder. Since the index and the secret key of the identified user are reconstructed at the decoder, the sum of the optimal values for the identification and secret-key rates is equal to this value, implying the optimal secret-key rate is $R_{S}=\frac{1}{2}\log\left(\frac{1}{\alpha \rho_{1}^{2}\rho_{2}^{2}+1-\rho_{1}^{2}\rho_{2}^{2}}\right)-R_{I}$. One can see that these rates are in a trade-off relation as the identification rate rises, the secret-key rate falls off. In the bottom one, $\frac{1}{2}\log\left(\frac{1}{\alpha }\right)$ is the entire rate that we need to generate auxiliary random sequences for encoding. The first part (blue part) represents the secret-key rate, and the second half $\left(\frac{1}{2}\log\left(\frac{1}{\alpha }\right)-R_{S}=\frac{1}{2}\log\left(\frac{\alpha \rho_{1}^{2}\rho_{2}^{2}+1-\rho_{1}^{2}\rho_{2}^{2}}{\alpha }\right)+R_{I}\right)$ is the rate of the sequences that are shared between the encoder and decoder to help estimation of the index and secret key, corresponding the storage rate. Storing the helper data at this rate results in leaking the user’s privacy at least $\frac{1}{2}\log\left(\frac{\alpha \rho_{1}^{2}\rho_{2}^{2}+1-\rho_{1}^{2}\rho_{2}^{2}}{\alpha \rho_{1}^{2}+1-\rho_{1}^{2}}\right)+R_{I}$, which is the optimal or minimum privacy-leakage for a given α.

For Figure 3b (the chosen-secret BIS model), the relation of the identification and secret-key rates is the same as in the generated-secret BIS model. However, the optimal storage rate becomes larger than the one seen in Figure 3a, equal to $\frac{1}{2}\log\left(\frac{1}{\alpha }\right)$ (the bottom band chart of Figure 3b), as the information related to the secret key chosen at the encoder (the concealed part) must be saved together with the helper data in DB to help the estimation of the key. For the privacy-leakage rate, the minimum values are not distinct in both models. This is because the unconcealed part of the storage at rate $\frac{1}{2}\log\left(\frac{1}{\alpha }\right)-R_{S}=\frac{1}{2}\log\left(\frac{\alpha \rho_{1}^{2}\rho_{2}^{2}+1-\rho_{1}^{2}\rho_{2}^{2}}{\alpha }\right)+R_{I}$, identical to the optimal storage rate of the generated-secret BIS model, is still exposed publicly, and thus the minimum privacy-leakage rates of the two models are the same.

**Figure 4:**
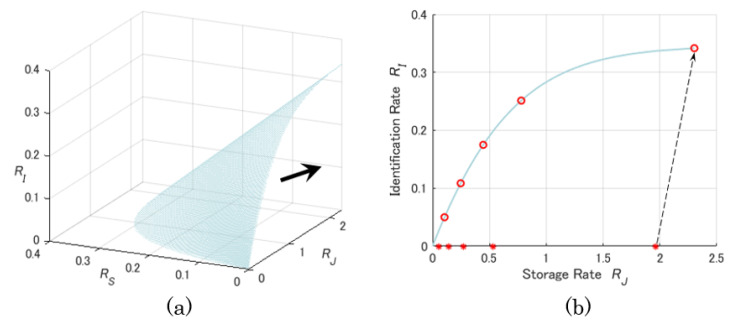
Projections of the capacity region $R_{G}$ onto (**a**) $R_{J}R_{S}R_{I}$-space and (**b**) $R_{J}R_{I}$-plane.

Figure 4 shows a numerical example of the region $R_{G}$ for $\rho_{1}^{2}=3/4$ and $\rho_{2}^{2}=2/3$. More specially, Figure 4a is a projection of the capacity region to the three-dimensional Euclidean space with X-axis $R_{J}$, Y-axis $R_{S}$, and Z-axis $R_{I}$. The black thick arrow indicates the direction of the achievable region for all rate tuples $\left(R_{J},R_{S},R_{I}\right)$. Figure 4b is another projection of the capacity region to $R_{J}R_{I}$-plane. Red asterisks and circles correspond to the rate points $\left(R_{J},R_{I}\right)$ at which $R_{I}$ is zero and $R_{I}$ is optimal, respectively, for some α∈(0,1]. To explain the relation of the identification and storage rates, let us focus on the rightmost asterisk and circle pair in Figure 4b. When identification rate varies from zero to the optimal value, the rate point $\left(R_{J},R_{I}\right)$ moves from the asterisk point (in the bottom) to the circled point along the arrow. From this, it is clear that the value of the storage rate for the circled point is greater compared to the asterisk point, implying that the change of identification rate affects the storage rate.

As a by-product of Theorem 1, the following corollary is obtained.

**Corollary** **1.** *The capacity regions of the generated- and chosen-secret BIS models with a single user (the models considered in* [8]*) for Gaussian sources are given by substituting $R_{I}=0$ into the right-hand sides of (19) and (20), respectively.*

**Remark** **3.** *Let $R_{G}^{\prime }$ and $R_{C}^{\prime }$ denote the capacity regions of the generated-secret and chosen-secret BIS models characterized via (11) in Remark 1. The two regions are provided below.*

(21) $$\begin{array}{cc} R_{G}^{\prime }=\underset{0<\alpha \leq 1}{⋃}\{\left(R_{I},R_{S},R_{J},R_{L}\right):R_{I}+R_{S} & \leq \frac{1}{2}\log\left(\frac{\left(\sigma_{x}^{2}+\sigma_{1}^{2}\right)\left(\sigma_{x}^{2}+\sigma_{2}^{2}\right)}{\alpha \sigma_{x}^{4}+\sigma_{x}^{2}\sigma_{1}^{2}+\sigma_{1}^{2}\sigma_{2}^{2}+\sigma_{2}^{2}\sigma_{x}^{2}}\right),\\ R_{J} & \geq \frac{1}{2}\log\left(\frac{\alpha \sigma_{x}^{4}+\sigma_{x}^{2}\sigma_{1}^{2}+\sigma_{1}^{2}\sigma_{2}^{2}+\sigma_{2}^{2}\sigma_{x}^{2}}{\alpha \left(\sigma_{x}^{2}+\sigma_{1}^{2}\right)\left(\sigma_{x}^{2}+\sigma_{2}^{2}\right)}\right)+R_{I},\\ R_{L} & \geq \frac{1}{2}\log\left(\frac{\alpha \sigma_{x}^{4}+\sigma_{x}^{2}\sigma_{1}^{2}+\sigma_{1}^{2}\sigma_{2}^{2}+\sigma_{2}^{2}\sigma_{x}^{2}}{\left(\alpha \sigma_{x}^{2}+\sigma_{1}^{2}\right)\left(\sigma_{x}^{2}+\sigma_{2}^{2}\right)}\right)+R_{I},\\ R_{I} & \geq 0,\;\;R_{S}\geq 0\}, \end{array}$$

(22) $$\begin{array}{cc} R_{C}^{\prime }=\underset{0<\alpha \leq 1}{⋃}\{\left(R_{I},R_{S},R_{J},R_{L}\right):R_{I}+R_{S} & \leq \frac{1}{2}\log\left(\frac{\left(\sigma_{x}^{2}+\sigma_{1}^{2}\right)\left(\sigma_{x}^{2}+\sigma_{2}^{2}\right)}{\alpha \sigma_{x}^{4}+\sigma_{x}^{2}\sigma_{1}^{2}+\sigma_{1}^{2}\sigma_{2}^{2}+\sigma_{2}^{2}\sigma_{x}^{2}}\right),\\ R_{J} & \geq \frac{1}{2}\log\left(\frac{1}{\alpha }\right),\\ R_{L} & \geq \frac{1}{2}\log\left(\frac{\alpha \sigma_{x}^{4}+\sigma_{x}^{2}\sigma_{1}^{2}+\sigma_{1}^{2}\sigma_{2}^{2}+\sigma_{2}^{2}\sigma_{x}^{2}}{\left(\alpha \sigma_{x}^{2}+\sigma_{1}^{2}\right)\left(\sigma_{x}^{2}+\sigma_{2}^{2}\right)}\right)+R_{I},\\ R_{I} & \geq 0,\;\;R_{S}\geq 0\}. \end{array}$$

*It can be verified that $R_{G}$ and $R_{C}$ are equivalent to $R_{G}^{\prime }$ and $R_{C}^{\prime }$, respectively, if one sets $\rho_{1}^{2}=\sigma_{x}^{2}/\left(\sigma_{x}^{2}+\sigma_{1}^{2}\right)$ and $\rho_{2}^{2}=\sigma_{x}^{2}/\left(\sigma_{x}^{2}+\sigma_{2}^{2}\right)$, respectively. In addition, as a connection to the result in a previous study, when there is no secret-key generation or provision $\left(R_{S}=0\right)$, and $R_{J},R_{L}$ are large enough ($R_{J},R_{L}\to \infty$), one can easily see that in $R_{G}^{\prime }$ and $R_{C}^{\prime }$, the maximum value of $R_{I}$ is $\frac{1}{2}\log\left(\frac{\left(\sigma_{x}^{2}+\sigma_{1}^{2}\right)\left(\sigma_{x}^{2}+\sigma_{2}^{2}\right)}{\sigma_{x}^{2}\sigma_{1}^{2}+\sigma_{1}^{2}\sigma_{2}^{2}+\sigma_{2}^{2}\sigma_{x}^{2}}\right)=\frac{1}{2}\log\left(1+\frac{\sigma_{x}^{2}}{\sigma_{1}^{2}+\sigma_{2}^{2}+\sigma_{1}^{2}\sigma_{2}^{2}/\sigma_{x}^{2}}\right)$. This value is exactly the identification capacity of the BIS for non-standard Gaussian RVs shown in* [5] *(Equation (21)) and it is achieved when α↓0.*

Another special case where $R_{I}=0$ (only one user), $R_{J}\to \infty$ (the storage rate is sufficiently large), and $\rho_{1}\to 1$ (the enrollment channel is noiseless), one can see that Theorem 1 naturally reduces to the characterizations of [23].

## 4. Behaviors of the Capacity Region

### 4.1. Optimal Asymptotic Rates and Zero-Rate Slopes

For the sake of succinct discussion, we concentrate on the generated-secret BIS model at which $R_{I}=0$, and the capacity region for this case is denoted by R, whose characterization is obtained by setting $R_{I}=0$ in the right-hand side of (19). We first investigate some special points of secret-key and privacy-leakage rates when storage rate becomes extremely low or large. Define two rate functions of $R_{J}$ as

(23) $$\begin{array}{c} R_{S}^{*}\left(R_{J}\right)=\max_{(R_{S},R_{J},R_{L})\in R}R_{S},\;\;\;\;\;\;\;\;\;\;\;\;\;\;\;\;\;\;\;R_{L}^{*}\left(R_{J}\right)=\min_{(R_{S},R_{J},R_{L})\in R}R_{L}, \end{array}$$

where the left and right equations in (23) are the maximum secret-key rate and the minimum privacy-leakage rate, respectively. Moreover, we define $R_{J}^{\alpha }=\frac{1}{2}\log\left(\frac{\alpha \rho_{1}^{2}\rho_{2}^{2}+1-\rho_{1}^{2}\rho_{2}^{2}}{\alpha }\right),$ so that we can write

(24) $$\begin{array}{cc} R_{S}^{*}\left(R_{J}^{\alpha }\right) & =\frac{1}{2}\log\left(\frac{1-\rho_{1}^{2}\rho_{2}^{2}/2^{2(R_{J}^{\alpha })}}{1-\rho_{1}^{2}\rho_{2}^{2}}\right),\\ R_{L}^{*}\left(R_{J}^{\alpha }\right) & =\frac{1}{2}\log\left(\frac{1-\rho_{1}^{2}\rho_{2}^{2}}{1-\rho_{1}^{2}+\rho_{1}^{2}\left(1-\rho_{2}^{2}\right)/2^{2(R_{J}^{\alpha })}}\right). \end{array}$$

As $R_{J}^{\alpha }\to \infty \;\left(\alpha ↓0\right)$, the optimal asymptotic secret-key rate and the amount of privacy-leakage approach to

(25) $$\lim_{R_{J}^{\alpha }\to \infty }R_{S}^{*}\left(R_{J}^{\alpha }\right)=\frac{1}{2}\log\left(\frac{1}{1-\rho_{1}^{2}\rho_{2}^{2}}\right)=I\left(Y;Z\right),$$

(26) $$\begin{array}{cc} \lim_{R_{J}^{\alpha }\to \infty }R_{L}^{*}\left(R_{J}^{\alpha }\right) & =\frac{1}{2}\log\left(\frac{1-\rho_{1}^{2}\rho_{2}^{2}}{1-\rho_{1}^{2}}\right)=\frac{1}{2}\log\left(\frac{1}{1-\rho_{1}^{2}}\right)-\frac{1}{2}\log\left(\frac{1}{1-\rho_{1}^{2}\rho_{2}^{2}}\right)\\ \; & =I(X;Y)-I(Z;Y). \end{array}$$

The result (25) corresponds to the optimal asymptotic secret-key rate [23] (Sect. III-B), and in order to achieve this value, it is required to let the storage rate go to infinity and leak the user’s privacy up to rate I(X;Y)−I(Z;Y).

In contrast, when $R_{J}↓0$, it is evident that $R_{S}$ and $R_{L}$ become zero as well, which does not carry much information. However, to investigate the BIS that achieves high secret-key and small privacy-leakage rates in the low storage rate regime, the zero-rate slopes of secret-key and privacy-leakage rates, namely, how fast these rates converge to zero, are important indicators. In light of (24), by a few steps of calculations, the slopes of secret-key and privacy-leakage rates at $R_{J}↓0$ can be determined as follows:

(27) $$\begin{array}{cc} \frac{dR_{S}^{*}\left(R_{J}^{\alpha }\right)}{dR_{J}^{\alpha }}{|}_{R_{J}^{\alpha }=0} & =\frac{\rho_{1}^{2}\rho_{2}^{2}}{1-\rho_{1}^{2}\rho_{2}^{2}}, \end{array}$$

(28) $$\begin{array}{cc} \frac{dR_{L}^{*}\left(R_{J}^{\alpha }\right)}{dR_{J}^{\alpha }}{|}_{R_{J}^{\alpha }=0} & =\frac{\rho_{1}^{2}\left(1-\rho_{2}^{2}\right)}{1-\rho_{1}^{2}\rho_{2}^{2}}=\frac{\rho_{1}^{2}\rho_{2}^{2}}{1-\rho_{1}^{2}\rho_{2}^{2}}\cdot \frac{1-\rho_{2}^{2}}{\rho_{2}^{2}}, \end{array}$$

where (27) is equal to the signal-to-noise ratio (SNR) of the channel from *Y* to *Z*, and this value multiplied by the reverse of the SNR of the channel $P_{Z|X}$ appears in the slope of privacy-leakage rate in (28).

### 4.2. Examples

Next, we give numerical computations of three different examples and take a look into behaviors of the special points.

Ex.1: (a) $\rho_{1}^{2}=3/4,\rho_{2}^{2}=2/3$, (b) $\rho_{1}^{2}=7/8,\rho_{2}^{2}=2/3$, (c) $\rho_{1}^{2}=15/16,\rho_{2}^{2}=2/3$,
Ex.2: (a) $\rho_{1}^{2}=3/4,\rho_{2}^{2}=2/3$, (b) $\rho_{1}^{2}=9/10,\rho_{2}^{2}=7/8$, (c) $\rho_{1}^{2}=15/16,\rho_{2}^{2}=11/12$,
Ex.3: (a) $\rho_{1}^{2}=3/4,\rho_{2}^{2}=2/3$, (b) $\rho_{1}^{2}=3/4,\rho_{2}^{2}=8/9$, (c) $\rho_{1}^{2}=3/4,\rho_{2}^{2}=14/15$.

**Figure 5:**
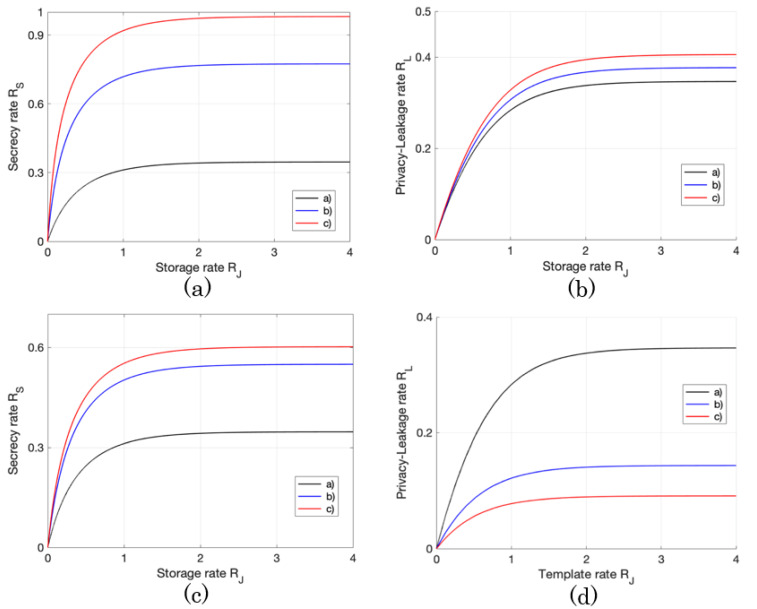
The projections of the capacity region $R_{G}$ onto two dimension figures for Exs. 2 and 3. (**a**) is the boundary of the capacity region $R_{G}$ onto $R_{J}R_{S}$-plane for Ex. 2. (**b**) is the boundary of the capacity region $R_{G}$ onto $R_{J}R_{L}$-plane for Ex. 2. (**c**) is the boundary of the capacity region $R_{G}$ onto $R_{J}R_{S}$-plane for Ex. 3. (**d**) is the boundary of the capacity region $R_{G}$ into $R_{J}R_{L}$-plane for Ex. 3.

Note that as $\rho_{1}^{2},\rho_{2}^{2}$ are large, the levels of noises (noises with smaller variances) added to the bio-data sequences at the encoder and decoder become small. Example 1 is the case where the level of noise at the encoder gradually decreases from (a) to (c), but the level of noise at the decoder stays constant for each round. Example 2 is the case in which the levels of noises at both the encoder and decoder are improved gradually from (a) to (c). Example 3 is opposite to Example 1. The calculated results of the secret-key and privacy-leakage rates for these cases are summarized in Table 1 and Table 2, and Figure 5.

It is ideal to keep the privacy-leakage rate small while producing a high secret-key rate, but Example 1 works out in the opposite way (cf. the rows of Ex. 1 in Table 1 and Table 2), so this is not a preferable choice. Example 2 realizes a high secret-key rate, but the amount of privacy-leakage remains high at some level, too (cf. the rows of Ex. 2 in Table 1 and Table 2, and Figure 5a,b). On the other hand, in Example 3, the privacy-leakage rate declines, but the secret-key rate becomes smaller compared to Example 2 (cf. the rows of Ex. 3 in Table 1 and Table 2, and Figure 5c,d). From these behaviors, we may conclude that it is unmanageable to achieve both high secret-key and small privacy-leakage rates at the same time. If one aims to achieve a high secret-key rate, it is important to diminish the levels of noises at both encoder and decoder, e.g., deploying quantizers with high quality, but this could result in leaking more users’ privacy. In different circumstances, to achieve a small privacy-leakage rate, it is preferable to maintain a certain level of noise at the encoder and pay sufficient attention for processing the noise’s level at the decoder. In this way, however, the gain of the secret-key rate may be dropped.

## 5. Overviews of the Proof of Theorem 1

The detailed proof of Theorem 1 is provided in Appendix A for $R_{G}$ and Appendix B for $R_{C}$. The regions $R_{G}$ and $R_{C}$ can be derived similarly, and the difference is that one-time pad is used to conceal the chosen secret key for secure transmission in the proof of $R_{C}$. The proof of each region consists of two parts: achievability and converse parts. The converse proof follows by applying Fano’s inequality [26], and the conditional version of EPI [27] doubly in two different directions. In the achievability part, the modified typical set [11], giving the so-called Markov lemma for weak typicality, helps us show that the error probability of the BIS vanishes since the so-called Markov lemma based on strong typicality can not be applied to the case of continuous RVs. Though a more general version of the Markov lemma for Gaussian sources, including lossy reconstruction, is shown in [28], we found that the two properties of the modified typical set are handy tools for checking all conditions in Definitions 1 and 2, and thus we provide our proof of the achievability based on this set. To evaluate the secret-key, secrecy-leakage, and privacy-leakage rates, we extend [29] (Lemma 4) to include continuous RVs so that the extended one can be used to derive the upper bounds on conditional differential entropies of jointly typical sequences, appearing in these evaluations.

## 6. Conclusions and Future Work

We characterized the capacity regions of identification, secret-key, storage, and privacy-leakage rates for both generated- and chosen-secret BIS models under Gaussian sources and channels. We showed that an idea for deriving the capacity regions of the BIS with HSM is to convert the system to another one, where the data flows of each user are in one-way direction. We also gave numerical computations of three different examples for the generated-secret BIS model, and from these results, it appeared that achieving high secret-key and small privacy-leakage rates simultaneously is unlikely manageable.

For future work, a natural extension is to characterize the capacity regions of the BIS with Gaussian vector sources and channels. In the scalar Gaussian case, we showed that it suffices to use a single parameter to characterize the optimal trade-off of the BIS. However, for Gaussian vector sources, the optimal trade-off regions is generally in the form of the covariance matrix optimization problem, and solving the problem becomes more challenging as one may need to use the enhancement technique, introduced in [30], to characterize the capacity regions.

Another extension is to construct practical codes that can achieve the capacity regions. In the BIS with a single user, convolutional and turbo codes that control the privacy-leakage were investigated in [31] and applied to real-life application, Electroencephalograph, in [32]. In these studies, it was shown that by applying vector quantization at the encoder and soft-decision at the decoder for Gaussian sources, a lower privacy-leakage rate was realizable. However, to the best of our knowledge, there has not yet been any studies dealing with practical codes for the BIS with multiple users. This remains as an interesting research topic.

## Figures and Tables

**Table 1:** The secret-key and privacy-leakage rates when $R_{J}\to \infty$.

Cases	The Optimal Secret-Key Rate			Privacy-Leakage Rate		
	(a)	(b)	(c)	(a)	(b)	(c)
Ex. 1	0.35	0.44	0.49	0.35	0.6	0.90
Ex. 2	0.35	0.77	0.98	0.35	0.38	0.41
Ex. 3	0.35	0.55	0.6	0.35	0.14	0.09

**Table 2:** The slopes of secret-key and privacy-leakage rates at $R_{J}↓0$.

Cases	The Slope of Secret-Key Rate			The Slope of Privacy-Leakage Rate		
	(a)	(b)	(c)	(a)	(b)	(c)
Ex. 1	1.0	1.40	1.67	0.5	0.7	0.83
Ex. 2	1.0	3.71	6.11	0.5	0.53	0.56
Ex. 3	1.0	2.0	2.33	0.5	0.25	0.17

**Figure A1:**
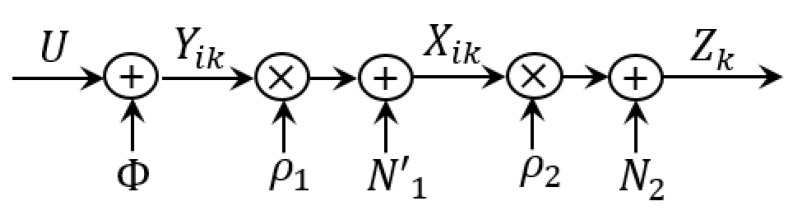
Relation among RVs (U,Y,X,Z).

**Figure A2:**
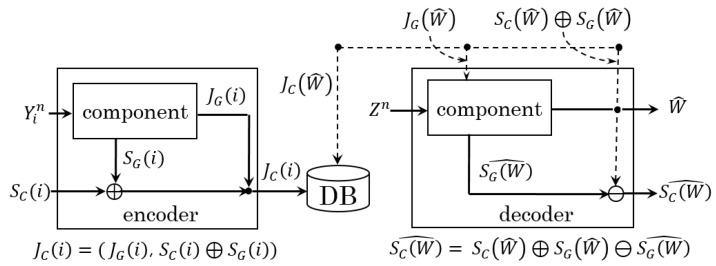
Encoder and decoder of the chosen-secret BIS model for achievability proof.

## Data Availability

Not applicable.

## Appendix A. Proof of Equation (19)

In this appendix, we give the proof of the capacity region of the generated-secret BIS model. Before proceeding to the proof, we review the definitions of the weakly typical and modified typical sets, and some properties of these sets.

### Appendix A.1. Weakly Typical Sets and Modified Typical Sets

The definition and property of weakly typical set hold for both discrete and continuous RVs, but here we provide only the continuous version.

**Definition A1.***(Weakly ϵ-typical set for continuous RVs* [26] *(Chapter 8))*
*Let $X_{1},X_{2},\cdots ,X_{k}$ be a sequence of continuous RVs drawn i.i.d. according to the joint pdf $f_{X_{1}X_{2}\cdots X_{k}}\left(x_{1},x_{2},\cdots ,x_{k}\right)$. For small enough ϵ>0, and any n, the weakly ϵ-typical set $A_{\epsilon }^{\left(n\right)}\left(X_{1}X_{2}\cdots X_{k}\right)$ with respect to $f_{X_{1}X_{2}\cdots X_{k}}\left(x_{1},x_{2},\cdots ,x_{k}\right)$ is defined as follows: *

(A1) $$\begin{array}{c} A_{\epsilon }^{\left(n\right)}\left(X_{1}X_{2}\cdots X_{k}\right)=\left\{\left(x_{1}^{n},x_{2}^{n},\cdots ,x_{k}^{n}\right):\left|-\frac{1}{n}\log f_{S^{n}}\left(s^{n}\right)-h\left(S\right)\right|<\epsilon ,\forall S\subseteq \left\{X_{1},X_{2},\cdots ,X_{k}\right\}\right\}, \end{array}$$

*where $s^{n}\subseteq \left\{x_{1}^{n},x_{2}^{n},\cdots ,x_{k}^{n}\right\}$ corresponding to RV S and $f_{S^{n}}\left(s^{n}\right)=\prod_{t=1}^{n}f_{S_{t}}\left(s_{t}\right)$. Moreover, the conditional ϵ-typical set is defined as $A_{\epsilon }^{\left(n\right)}\left(X_{k}|x_{2}^{n},\cdots ,x_{k-1}^{n}\right)=\left\{x_{k}^{n}:\left(x_{1}^{n},x_{2}^{n},\cdots ,x_{k}^{n}\right)\in A_{\epsilon }^{\left(n\right)}\left(X_{1}X_{2}\cdots X_{k}\right)\right\}.$*

The weakly ϵ-typical set $A_{\epsilon }^{\left(n\right)}\left(\cdot \right)$ has the following properties.

**Lemma** **A1.** *(Some properties of weakly ϵ-typical set* [26]*)*

1 *For $\forall S\subseteq \{X_{1},X_{2},\cdots ,X_{k}\}$ and large enough n,*
  (A2) $$\begin{array}{cc} \mathrm{Pr}\{A_{\epsilon }^{\left(n\right)}\left(S\right)\} & \geq 1-\epsilon . \end{array}$$
2 *For $\forall S,V\subseteq \{X_{1},X_{2},\cdots ,X_{k}\}$ (S∩V=Ø), we have that*
  (A3) $$\begin{array}{c} \mathrm{Vol}\left(A_{\epsilon }^{\left(n\right)}\left(V|s^{n}\right)\right)\leq e^{n(h(V|S)+2\epsilon )}, \end{array}$$
  *where Vol(·) denotes the volume of a set.*
3 *Fix k=2. If $\left({\tilde{X}}_{1}^{n},{\tilde{X}}_{2}^{n}\right)$ are independent sequences with the same marginals as $f_{X_{1}^{n}X_{2}^{n}}\left(x_{1}^{n},x_{2}^{n}\right)$, then*
  (A4) $$\begin{array}{c} \mathrm{Pr}\left\{\left({\tilde{X}}_{1}^{n},{\tilde{X}}_{2}^{n}\right)\in A_{\epsilon }^{\left(n\right)}\left(X_{1}X_{2}\right)\right\}\leq e^{-n(I\left(X_{1};X_{2}\right)-2\epsilon )}. \end{array}$$
  *Moreover, for n large enough,*
  (A5) $$\begin{array}{c} \mathrm{Pr}\left\{\left({\tilde{X}}_{1}^{n},{\tilde{X}}_{2}^{n}\right)\in A_{\epsilon }^{\left(n\right)}\left(X_{1}X_{2}\right)\right\}\geq \left(1-\epsilon \right)e^{-n(I\left(X_{1};X_{2}\right)+2\epsilon )}. \end{array}$$

**Proof.** See [26] (Section 15.2). □

Next, we provide the definition of the modified ϵ-typical set. This set gives the so-called Markov lemma for the weak typicality.

**Definition A2.***(Modified ϵ-typical set* [11] *(Appendix A-A))*
*Consider that (X,Y,U) forms a Markov chain X−Y−U, i.e., $f_{XYU}\left(x,y,u\right)=f_{XY}\left(x,y\right)f_{U|Y}\left(u|y\right)$. The modified ϵ-typical set $B_{\epsilon }^{\left(n\right)}\left(YU\right)$ is defined as*

(A6) $$\begin{array}{c} B_{\epsilon }^{\left(n\right)}\left(YU\right)=\left\{\left(y^{n},u^{n}\right):\mathrm{Pr}\left\{X^{n}\in A_{\epsilon }^{\left(n\right)}\left(X|y^{n},u^{n}\right)|\left(Y^{n},U^{n}\right)=\left(y^{n},u^{n}\right)\right\}\geq 1-\epsilon \right\}, \end{array}$$

*where $X^{n}$ is drawn i.i.d. from the transition probability $\prod_{k=1}^{n}f_{X|Y}\left(x_{k}|y_{k}\right)$. In addition, define $B_{\epsilon }^{\left(n\right)}\left(U|y^{n}\right)=\left\{u^{n}:\left(y^{n},u^{n}\right)\in B_{\epsilon }^{\left(n\right)}\left(YU\right)\right\}$ for all $y^{n}$, and $B_{\epsilon }^{\left(n\right)}{\left(U|y^{n}\right)}^{c}$ denotes the complementary set of $B_{\epsilon }^{\left(n\right)}\left(U|y^{n}\right)$.*

The modified set induces two useful properties below.

**Lemma** **A2.** *(Properties of the modified set* [11] *(Appendix A-A))*

Property 1. *If $\left(y^{n},u^{n}\right)\in B_{\epsilon }^{\left(n\right)}\left(YU\right)$ then also $\left(y^{n},u^{n}\right)\in A_{\epsilon }^{\left(n\right)}\left(YU\right)$.*
Property 2. *Assume that $\left(U^{n},Y^{n},X^{n}\right)∼f_{U^{n}X^{n}Y^{n}}=\prod_{t=1}^{n}f_{X_{t}Y_{t}}f_{U_{t}|Y_{t}}$. Then, for ϵ∈(0,1) and n large enough, $\sum_{\left(y^{n},u^{n}\right)\in B_{\epsilon }^{\left(n\right)}\left(YU\right)}P_{Y^{n}U^{n}}\left(y^{n},u^{n}\right)\geq 1-\epsilon$.*

**Proof.** See [11] (Appendix A-A). □

Now we are at the position to present the detailed proofs of Equation (19).

### Appendix A.2. Achievability Part

Let 0<α≤1 and fix δ>0 (small enough positive), the block length *n*, and the joint pdf of (U,Y,X,Z) such that the Markov chain U−Y−X−Z holds. Let U∼N(0,1−α), Gaussian RV with mean zero and variance 1−α. As shown in Figure A1, based on the converted system, consider that $Y_{ik}=U+\Phi ,$ where Φ∼N(0,α), independent of *U*, is Gaussian with mean zero and variance α. From (9) and (10) of the converted system, it holds that

(A7) $$\begin{array}{c} X_{ik}=\rho_{1}U+\rho_{1}\Phi +N_{1}^{\prime },\;\;\;\;\;\;\;\;\;\;Z_{k}=\rho_{1}\rho_{2}U+\rho_{1}\rho_{2}\Phi +\rho_{2}N_{1}^{\prime }+N_{2}. \end{array}$$

Hence, we readily see that

(A8) $$\begin{array}{cc} I(Y;U) & =\frac{1}{2}\log\left(\frac{1}{\alpha }\right),\;\;\;\;\;\;I\left(X;U\right)=\frac{1}{2}\log\left(\frac{1}{\alpha \rho_{1}^{2}+1-\rho_{1}^{2}}\right),\\ I(Z;U) & =\frac{1}{2}\log\left(\frac{1}{\alpha \rho_{1}^{2}\rho_{2}^{2}+1-\rho_{1}^{2}\rho_{2}^{2}}\right). \end{array}$$

Now set $0<R_{I}<I\left(Z;U\right)$, and

(A9) $$\begin{array}{cc} R_{S} & =I\left(Z;U\right)-R_{I}-2\delta ,\;\;\;\;\;\;R_{J}=I\left(Y;U\right)-I\left(Z;U\right)+R_{I}+6\delta , \end{array}$$

(A10) $$\begin{array}{cc} R_{L} & =I\left(X;U\right)-I\left(Z;U\right)+R_{I}+6\delta ,\;\;\;\;\;\;M_{I}=2^{nR_{I}},\;\;\;\;\;\;M_{S}=2^{nR_{S}},\;\;\;\;\;\;M_{J}=2^{nR_{J}}, \end{array}$$

where the values of I(Y;U),I(X;U), and I(Z;U) are specified in (A8). Also, recall that $I=\left[1:M_{I}\right],S=\left[1:M_{S}\right],\;J=\left[1:M_{J}\right]$.

Next we generate $e^{n(I(Y;U)+\delta )}$ sequences of $u^{n}\left(s,j\right)$ i.i.d. from pdf $f_{U}$, where s∈S and j∈J.

Seeing $y_{i}^{n}\;\left(i\in I\right)$, the encoder finds $u^{n}\left(s,j\right)$ such that $\left(y_{i}^{n},u^{n}\left(s,j\right)\right)\in B_{\delta }^{\left(n\right)}\left(YU\right)$, denoting the modified set defined in Definition A2. If there are multiple pairs of such (s,j), the encoder picks one at random. If there are no such pairs, it declares error. We denote the chosen pair as (s(i),j(i)), where each element is a function of the index *i*. The helper j(i) is stored in the helper DB and secret key s(i) is saved in the key DB at location *i*, respectively.

Observing $z^{n}$, the noisy sequence of the identified user $x_{w}^{n}$, the decoder looks for $u^{n}\left(s,j\left(i\right)\right)$ such that $\left(z^{n},u^{n}\left(s,j\left(i\right)\right)\right)\in A_{\delta }^{\left(n\right)}\left(ZU\right)$ for some i∈I and s∈S, where $A_{\delta }^{\left(n\right)}\left(ZU\right)$ denotes the weakly δ-typical set. If a unique pair (i,s) is found, it outputs $\left(\hat{w},\hat{s(w)}\right)=\left(i,s\right)$, or else it declares error.

Let (S(i),J(i)) denote the index pair chosen at the encoder based on $Y_{i}^{n}$, i.e., $\left(Y_{i}^{n},U^{n}\left(S\left(i\right),J\left(i\right)\right)\right)\in B_{\delta }^{\left(n\right)}\left(YU\right)$. Furthermore, we denote $U^{n}\left(S\left(i\right),J\left(i\right)\right)$ as $U_{i}^{n}$ for notational simplicity. Note that the pair (S(i),J(i)) can determine the sequence $U_{i}^{n}$ precisely. Next, we check all conditions in Definition 1 hold for a random codebook $C_{n}=\{U^{n}\left(s,j\right),\;s\in S$ and j∈J}.

*Analysis of Error Probability*: For W=i, an error event possibly happens at the encoder is:

ℰ1 : {$\left(Y_{i}^{n},U^{n}\left(s,j\right)\right)\notin B_{\delta }^{\left(n\right)}\left(YU\right)$ for all s∈S and j∈J},

and those at the decoder are:

ℰ2 : {$\left(Z^{n},U_{i}^{n}\right)\notin A_{\delta }^{\left(n\right)}\left(ZU\right)$},
ℰ3 : {$(Z^{n},U^{n}\left(s^{\prime },J\left(i\right)\right))\in A_{\delta }^{\left(n\right)}\left(ZU\right)$ for some $s^{\prime }\in S,\;s^{\prime }\neq S\left(i\right)$},
ℰ4 : {$(Z^{n},U^{n}\left(s,J\left(i^{\prime }\right)\right))\in A_{\delta }^{\left(n\right)}\left(ZU\right)$ for some $i^{\prime }\in I,\;i^{\prime }\neq i$, and s∈S}.

As usual, we use the random coding argument, and the ensemble average of the error probability can be evaluated as

(A11) $$\begin{array}{cc} \mathrm{Pr}\{\left(\hat{W},\hat{S(W)}\right)\neq \left(W,S\left(W\right)\right)|W=i\} & =\mathrm{Pr}\{E_{1}\cup E_{2}\cup E_{3}\cup E_{4}\}\\ \; & \leq \mathrm{Pr}\left\{E_{1}\right\}+\mathrm{Pr}\left\{E_{2}\cap E_{1}^{c}\right\}+\mathrm{Pr}\left\{E_{3}\right\}+\mathrm{Pr}\left\{E_{4}\right\}, \end{array}$$

where each of Pr{·} on the right-hand side denotes the conditional probability given W=i.

Now let us focus on bounding each term individually. The first term of (A11) can be made arbitrarily small by a similar argument of [11] (cf. the analysis of First Term of Error Probability in Appendix A-B). The rest can be analyzed as follows. For the second term, it follows that

(A12) $$\begin{array}{cc} \mathrm{Pr}\left\{E_{2}\cap E_{1}^{c}\right\}\; & =\mathrm{Pr}\{\left(Z^{n},U_{i}^{n}\right)\notin A_{\delta }^{\left(n\right)}\left(ZU\right)\cap \left(Y_{i}^{n},U_{i}^{n}\right)\in B_{\delta }^{\left(n\right)}\left(YU\right)\}\\ \; & \leq \mathrm{Pr}\{\left(Z^{n},Y_{i}^{n},U_{i}^{n}\right)\notin A_{\delta }^{\left(n\right)}\left(ZYU\right)\cap \left(Y_{i}^{n},U_{i}^{n}\right)\in B_{\delta }^{\left(n\right)}\left(YU\right)\}\\ \; & ={\;\int \int \;}_{B_{\delta }^{\left(n\right)}\left(YU\right)}f_{Y_{i}^{n}U_{i}^{n}}\left(y^{n},u^{n}\right)\mathrm{Pr}\left\{Z^{n}\notin A_{\delta }^{\left(n\right)}\left(Z|y^{n},u^{n}\right)|\left(Y_{i}^{n},U_{i}^{n}\right)=\left(y^{n},u^{n}\right)\right\}d\left(y^{n},u^{n}\right)\\ \; & \overset{\left(a\right)}{\leq }\delta {\;\int \int \;}_{B_{\delta }^{\left(n\right)}\left(YU\right)}f_{Y_{i}^{n}U_{i}^{n}}\left(y^{n},u^{n}\right)d\left(y^{n},u^{n}\right)\leq \delta , \end{array}$$

where (a) follows from the definition of the modified δ-typical set due to the Markov chain Z−Y−U. To bound $\mathrm{Pr}\left\{E_{3}\right\}$, due to the symmetry in the codebook generation, we can set J(i)=1 and have that

(A13) $$\begin{array}{cc} \mathrm{Pr}\left\{E_{3}\right\} & =\mathrm{Pr}\{\left(Z^{n},U^{n}\left(s^{\prime },1\right)\right)\in A_{\delta }^{\left(n\right)}\left(ZU\right)\;\;\mathrm{for}\;\mathrm{some}\;s^{\prime }\in S,\;s^{\prime }\neq S\left(i\right)\}\\ \; & =\sum_{s\in S}\left(\mathrm{Pr}\left\{S\left(i\right)=s\right\}\cdot \mathrm{Pr}\{\left(Z^{n},U^{n}\left(s^{\prime },1\right)\right)\in A_{\delta }^{\left(n\right)}\left(ZU\right)\;\;\mathrm{for}\;\mathrm{some}\;s^{\prime }\in S,\;s^{\prime }\neq s|S\left(i\right)=s\}\right)\\ \; & =\sum_{s\in S}\left(\mathrm{Pr}\left\{S\left(i\right)=s\right\}\cdot \mathrm{Pr}\left\{\underset{s^{\prime }\in S\setminus s}{⋃}\left(Z^{n},U^{n}\left(s^{\prime },1\right)\right)\in A_{\delta }^{\left(n\right)}\left(ZU\right)|S\left(i\right)=s\right\}\right)\\ \; & \leq \sum_{s\in S}\left(\mathrm{Pr}\left\{S\left(i\right)=s\right\}\cdot \left(\sum_{s^{\prime }\in S\setminus s}\mathrm{Pr}\left\{\left(Z^{n},U^{n}\left(s^{\prime },1\right)\right)\in A_{\delta }^{\left(n\right)}\left(ZU\right)|S\left(i\right)=s\right\}\right)\right)\\ \; & \overset{\left(b\right)}{=}\sum_{s\in S}\left(\mathrm{Pr}\left\{S\left(i\right)=s\right\}\cdot \left(\sum_{s^{\prime }\in S\setminus s}\mathrm{Pr}\left\{\left(Z^{n},U^{n}\left(s^{\prime },1\right)\right)\in A_{\delta }^{\left(n\right)}\left(ZU\right)\right\}\right)\right)\\ \; & \leq \sum_{s\in S}\left(\mathrm{Pr}\left\{S\left(i\right)=s\right\}\cdot \left(\sum_{s^{\prime }\in S\setminus s}e^{-n(I(Z;U)-\delta )}\right)\right)\\ \; & \leq M_{S}\cdot e^{-n(I(Z;U)-\delta )}\leq e^{-n\delta }, \end{array}$$

where (b) holds because the event $\left\{\left(Z^{n},U^{n}\left(s^{\prime },1\right)\right)\in A_{\delta }^{\left(n\right)}\left(ZU\right)\right\}$, $s^{\prime }\in S\setminus s$ and the event {S(i)=s} are mutually independent, and the last inequality in (A13) follows as $R_{S}<I\left(Z;U\right)$.

To evaluate $\mathrm{Pr}\left\{E_{4}\right\}$, we define two new events $E_{4}^{\prime }=\{\left(Z^{n},U^{n}\left(S\left(i\right),J\left(i^{\prime }\right)\right)\right)\in A_{\delta }^{\left(n\right)}\left(ZU\right)$ for some $i^{\prime }\neq i,\;i^{\prime }\in I\}$ and $E_{4}^{\prime \prime }=\{\left(Z^{n},U^{n}\left(s^{\prime },J\left(i^{\prime }\right)\right)\right)\in A_{\delta }^{\left(n\right)}\left(ZU\right)$ for some $i^{\prime }\neq i,\;i^{\prime }\in I$ and $s^{\prime }\neq S\left(i\right),\;s^{\prime }\in S\}$. Because $E^{\prime }\cap E^{\prime \prime }=Ø$, it follows that $\mathrm{Pr}\left\{E_{4}\right\}=\mathrm{Pr}\left\{E_{4}^{\prime }\right\}+\mathrm{Pr}\left\{E_{4}^{\prime \prime }\right\}$. For $\mathrm{Pr}\left\{E_{4}^{\prime }\right\}$, without loss of generality, we set S(i)=1. Then, it follows that

(A14) $$\begin{array}{cc} \mathrm{Pr}\left\{E_{4}^{\prime }\right\} & =\mathrm{Pr}\{\left(Z^{n},U^{n}\left(1,J\left(i^{\prime }\right)\right)\right)\in A_{\delta }^{\left(n\right)}\left(ZU\right)\;\;\mathrm{for}\;\mathrm{some}\;i^{\prime }\in I,\;i^{\prime }\neq i\}\\ \; & \leq \sum_{i^{\prime }\in I\setminus i}\mathrm{Pr}\left\{\left(Z^{n},U^{n}\left(1,J\left(i^{\prime }\right)\right)\right)\in A_{\delta }^{\left(n\right)}\left(ZU\right)\right\}\\ \; & \leq \sum_{i^{\prime }\in I\setminus i}e^{-n(I(Z;U)-\delta )}\leq M_{I}\cdot e^{-n(I(Z;U)-\delta )}\leq e^{-n\delta }, \end{array}$$

where the last inequality in (A14) follows as $R_{I}<I\left(Z;U\right)$. For $\mathrm{Pr}\left\{E_{4}^{\prime \prime }\right\}$, due to the statistical independence of $Z^{n}$ and $U^{n}\left(s^{\prime },J\left(i^{\prime }\right)\right)$, we have that

(A15) $$\begin{array}{cc} \mathrm{Pr}\left\{E_{4}^{\prime \prime }\right\}\; & =\mathrm{Pr}\{\left(Z^{n},U^{n}\left(s^{\prime },J\left(i^{\prime }\right)\right)\right)\in A_{\delta }^{\left(n\right)}\left(ZU\right)\;\;\mathrm{for}\;\mathrm{some}\;i^{\prime }\neq i,\;i^{\prime }\in I\;\mathrm{and}\;s^{\prime }\neq S\left(i\right),\;s^{\prime }\in S\}\\ \; & =\mathrm{Pr}\left\{\underset{\begin{array}{c} i^{\prime }\in I\setminus i,\;s^{\prime }\in S\setminus S\left(i\right) \end{array}}{⋃}\left(Z^{n},U^{n}\left(s^{\prime },J\left(i^{\prime }\right)\right)\right)\in A_{\delta }^{\left(n\right)}\left(ZU\right)\right\}\\ \; & =\sum_{s\in S}\left(\mathrm{Pr}\left\{S\left(i\right)=s\right\}\cdot \mathrm{Pr}\left\{\underset{\begin{array}{c} i^{\prime }\in I\setminus i,\;s^{\prime }\in S\setminus s \end{array}}{⋃}\left(Z^{n},U^{n}\left(s^{\prime },J\left(i^{\prime }\right)\right)\right)\in A_{\delta }^{\left(n\right)}\left(ZU\right)|S\left(i\right)=s\right\}\right)\\ \; & \leq \sum_{s\in S}\left(\mathrm{Pr}\left\{S\left(i\right)=s\right\}\cdot \left(\sum_{i^{\prime }\in I\setminus i}\sum_{s^{\prime }\in S\setminus s}\mathrm{Pr}\left\{\left(Z^{n},U^{n}\left(s^{\prime },J\left(i^{\prime }\right)\right)\right)\in A_{\delta }^{\left(n\right)}\left(ZU\right)|S\left(i\right)=s\right\}\right)\right)\\ \; & =\sum_{s\in S}\left(\mathrm{Pr}\left\{S\left(i\right)=s\right\}\cdot \left(\sum_{i^{\prime }\in I\setminus i}\sum_{s^{\prime }\in S\setminus s}\mathrm{Pr}\left\{\left(Z^{n},U^{n}\left(s^{\prime },J\left(i^{\prime }\right)\right)\right)\in A_{\delta }^{\left(n\right)}\left(ZU\right)\right\}\right)\right)\\ \; & =\sum_{s\in S}\left(\mathrm{Pr}\left\{S\left(i\right)=s\right\}\cdot \left(\sum_{i^{\prime }\in I\setminus i}\sum_{s^{\prime }\in S\setminus s}e^{-n(I(Z;U)-\delta )}\right)\right)\\ \; & \leq M_{I}\cdot M_{S}\cdot e^{-n(I(Z;U)-\delta )}=e^{-n\delta }, \end{array}$$

where the last equality in (A15) holds as $\frac{1}{n}\log M_{I}+\frac{1}{n}\log M_{S}=I\left(Z;U\right)-2\delta$. Hence, the error probability is bounded by

(A16) $$\begin{array}{cc} \mathrm{Pr} & \{\left(\hat{W},\hat{S(W)}\right)\neq \left(W,S\left(W\right)\right)|W=i\}\leq 4\delta \end{array}$$

for large enough *n*.

Before diving into the detailed analysis, we state lemmas that are important for the rest of the evaluations.

**Lemma** **A3.**
*For given $u^{n}$ and large enough n, we have that*

(A17) $$\begin{array}{cc} \mathrm{Vol}\left(B_{\delta }^{\left(n\right)}\left(Y|u^{n}\right)\right) & \leq \mathrm{Vol}\left(A_{\delta }^{\left(n\right)}\left(Y|u^{n}\right)\right). \end{array}$$

**Proof.** This is a straightforward result from Property 1 of Lemma A2. □

The following lemma plays an essential role in bounding the secret-key, secrecy-leakage, and privacy-leakage rates of the BIS. Again recall that the index pair (S(i),J(i)) determines $U_{i}^{n}$ directly, and therefore Lemma A4 can be thought of an extended version of [29] (Lemma 4) to incorporate continuous RVs. In [29], the lemma was proved by using the strongly typical set [33], and the literature, e.g., [8,13] demonstrated that it could be finely applied to establish the achievability part of the BIS under noisy enrollment for DMS settings. However, when the sources of the BIS becomes Gaussian, it is not trivial whether the claim of this lemma still holds or not. Here, we provide a full proof of the extended version of [29] (Lemma 4) for Gaussian RVs by using the connection between the modified δ-typical set $B_{\delta }^{\left(n\right)}\left(\cdot \right)$ and the weakly δ-typical set $A_{\delta }^{\left(n\right)}\left(\cdot \right)$.

**Lemma** **A4.**
*It holds that*

(A18) $$\begin{array}{cc} h(Y_{i}^{n}|S\left(i\right),J\left(i\right),C_{n}) & \leq n(h\left(Y|U\right)+\delta_{n}), \end{array}$$

(A19) $$\begin{array}{cc} h(Y_{i}^{n}|X_{i}^{n},S\left(i\right),J\left(i\right),C_{n}) & \leq n(h\left(Y|X,U\right)+\delta_{n}), \end{array}$$

*where $\delta_{n}↓0$ as δ↓0 and n→∞.*

**Proof.** We first prove (A18). Define a binary RV *T* taking value 1 if $\left(Y_{i}^{n},U_{i}^{n}\right)\in B_{\delta }^{\left(n\right)}\left(YU\right)$, and 0 otherwise. In the analysis of the error probability, it is guaranteed that $P_{T}\left(0\right)\leq \delta$, i.e., $\left(Y_{i}^{n},U_{i}^{n}\right)\in B_{\delta }^{\left(n\right)}\left(YU\right)$ with high probability. The left-hand side of (A18) can be bounded as

(A20) $$\begin{array}{cc} h(Y_{i}^{n}|S\left(i\right),J\left(i\right),C_{n})\; & \overset{\left(c\right)}{=}h\left(Y_{i}^{n}|U_{i}^{n},S\left(i\right),J\left(i\right),C_{n}\right)\\ \; & \overset{\left(d\right)}{\leq }h\left(Y_{i}^{n}|U_{i}^{n}\right)\leq h\left(Y_{i}^{n},T|U_{i}^{n}\right)\leq H\left(T\right)+h\left(Y_{i}^{n}|U_{i}^{n},T\right)\\ \; & \leq 1+P_{T}\left(0\right)h\left(Y_{i}^{n}|U_{i}^{n},T=0\right)+P_{T}\left(1\right)h\left(Y_{i}^{n}|U_{i}^{n},T=1\right)\\ \; & \overset{\left(e\right)}{\leq }n\epsilon_{n}+h\left(Y_{i}^{n}|U_{i}^{n},T=1\right)\\ \; & =n\epsilon_{n}+\int_{R^{n}}h\left(Y_{i}^{n}|U_{i}^{n}=u^{n},T=1\right)dF\left(u^{n}\right)\\ \; & =n\epsilon_{n}+\int_{R^{n}}\int_{B_{\delta }^{\left(n\right)}\left(Y|u^{n}\right)}P_{Y_{i}^{n}|U_{i}^{n},T}\left(y^{n}|u^{n},1\right)\log\frac{1}{P_{Y_{i}^{n}|U_{i}^{n},T}\left(y^{n}|u^{n},1\right)}dy^{n}dF\left(u^{n}\right)\\ \; & \overset{\left(f\right)}{\leq }n\epsilon_{n}+\int_{R^{n}}\log\left(\int_{B_{\delta }^{\left(n\right)}\left(Y|u^{n}\right)}P_{Y_{i}^{n}|U_{i}^{n},T}\left(y^{n}|u^{n},1\right)\frac{1}{P_{Y_{i}^{n}|U_{i}^{n},T}\left(y^{n}|u^{n},1\right)}dy^{n}\right)dF\left(u^{n}\right)\\ \; & =n\epsilon_{n}+\int_{R^{n}}\log\left(\int_{B_{\delta }^{\left(n\right)}\left(Y|u^{n}\right)}dy^{n}\right)dF\left(u^{n}\right)\\ \; & =n\epsilon_{n}+\int_{R^{n}}\log\left(\mathrm{Vol}\left(B_{\delta }^{\left(n\right)}\left(Y|u^{n}\right)\right)\right)dF\left(u^{n}\right)\\ \; & \overset{\left(g\right)}{\leq }n\epsilon_{n}+\int_{R^{n}}\log\left(\mathrm{Vol}\left(A_{\delta }^{\left(n\right)}\left(Y|u^{n}\right)\right)\right)dF\left(u^{n}\right)\\ \; & \overset{\left(h\right)}{\leq }n\epsilon_{n}+n\left(h\left(Y|U\right)+\delta \right))\int_{R^{n}}dF\left(u^{n}\right)\\ \; & =n(h\left(Y|U\right)+\delta +\epsilon_{n}), \end{array}$$

where

(c) follows as (S(i),J(i)) determines $U_{i}^{n}$,
(d) follows because conditioning reduces entropy,
(e) follows as $h\left(Y_{i}^{n}|U_{i}^{n},T=0\right)\leq h\left(Y_{i}^{n}\right)=\frac{n}{2}\log\left(2\pi e\right)$, and we define $\epsilon_{n}=\frac{1}{n}+\frac{\delta }{2}\log\left(2\pi e\right)$,
(f) follows by applying Jensen’s inequality to the concave function ϕ(t)=−tlogt,
(g) is due to (A17) in Lemma A3,
(h) is due to (A3) in Lemma A1.

Therefore, from (A20), we obtain that

(A21) $$\begin{array}{cc} \frac{1}{n}h\left(Y_{i}^{n}|S\left(i\right),J\left(i\right),C_{n}\right) & \leq h\left(Y|U\right)+\delta_{n}, \end{array}$$

where $\delta_{n}=\delta +\epsilon_{n}$ and $\delta_{n}↓0$ as n→∞ and δ↓0. Next, we briefly summarize how to show (A19). The left-hand side of the inequality can be developed as $h\left(Y_{i}^{n}|X_{i}^{n},S\left(i\right),J\left(i\right),C_{n}\right)=h\left(Y_{i}^{n}|X_{i}^{n},U_{i}^{n},S\left(i\right),J\left(i\right),C_{n}\right)\leq h\left(Y_{i}^{n}|X_{i}^{n},U_{i}^{n}\right)$, where the equality and inequality follow due to the same reasons of (c) and (d) in (A20), respectively. By the definition of the modified δ-typical set in Definition A2, it can be concluded that $\mathrm{Pr}\{\left(X_{i}^{n},Y_{i}^{n},U_{i}^{n}\right)\in A_{\delta }^{\left(n\right)}\left(XYU\right)\}\to 1$ as n→∞ due to the Markov chain X−Y−U and $\left(Y_{i}^{n},U_{i}^{n}\right)\in B_{\delta }^{\left(n\right)}\left(YU\right)$ with high probability. This implies $\mathrm{Pr}\{\left(X_{i}^{n},U_{i}^{n}\right)\in A_{\delta }^{\left(n\right)}\left(XU\right)\}\to 1$ and $\mathrm{Pr}\{Y_{i}^{n}\in A_{\delta }^{\left(n\right)}\left(Y|x^{n},u^{n}\right)|\left(X_{i}^{n},U_{i}^{n}\right)=\left(x^{n},u^{n}\right)\}\to 1$ as n→∞ as well. Based on this observation, the rest of the proof for (A19) can be done similarly by the arguments seen in [29] (Appendix C), and therefore the details are omitted. □

Note that Equations (14) and (16) obviously hold from the parameter settings. By applying Lemma A4, bounds on the secret-key and the secrecy-leakage rates can be derived as follows:

(A22) $$\begin{array}{cc} \frac{1}{n}H\left(S\left(i\right)|C_{n}\right) & \geq R_{S}-5\delta =\frac{1}{n}\log M_{S}-5\delta ,\;\;\;\;\;\;\;\frac{1}{n}I\left(S\left(i\right);J\left(i\right)|C_{n}\right)\leq 5\delta \end{array}$$

for large enough *n*. For detailed discussions, the readers may refer to [14] (Proof of Theorem 1). *Analysis of Privacy-Leakage Rate*: From the left-hand side of (18), we have that

(A23) $$\begin{array}{cc} I(X_{i}^{n};J\left(i\right)|C_{n}) & =H\left(J\left(i\right)|C_{n}\right)-H\left(J\left(i\right)|X_{i}^{n},C_{n}\right)\\ \; & \leq nR_{J}-H\left(J\left(i\right)|X_{i}^{n},C_{n}\right)\\ \; & =n\left(I\left(Y;U\right)-I\left(Z;U\right)+R_{I}+6\delta \right)-H\left(J\left(i\right)|X_{i}^{n},C_{n}\right)\\ \; & =n\left(h\left(U|Z\right)-h\left(U|Y\right)+R_{I}+6\delta \right)-H\left(J\left(i\right)|X_{i}^{n},C_{n}\right). \end{array}$$

The last term in (A23) can be further evaluated as

(A24) $$\begin{array}{cc} H(J\left(i\right)|X_{i}^{n},C_{n})\; & =h\left(Y_{i}^{n},J\left(i\right)|X_{i}^{n},C_{n}\right)-h\left(Y_{i}^{n}|X_{i}^{n},J\left(i\right),C_{n}\right)\\ \; & =h\left(Y_{i}^{n},J\left(i\right)|X_{i}^{n},C_{n}\right)-h\left(Y_{i}^{n}|X_{i}^{n},J\left(i\right),S\left(i\right),C_{n}\right)-I\left(Y_{i}^{n};S\left(i\right)|X_{i}^{n},J\left(i\right),C_{n}\right)\\ \; & \overset{\left(i\right)}{=}h\left(Y_{i}^{n}|X_{i}^{n},C_{n}\right)-h\left(Y_{i}^{n}|X_{i}^{n},J\left(i\right),S\left(i\right),C_{n}\right)-H\left(S\left(i\right)|X_{i}^{n},J\left(i\right),C_{n}\right)\\ \; & \overset{\left(j\right)}{=}nh\left(Y|X\right)-h\left(Y_{i}^{n}|X_{i}^{n},J\left(i\right),S\left(i\right),C_{n}\right)-H\left(S\left(i\right)|X_{i}^{n},J\left(i\right),Z^{n},C_{n}\right)\\ \; & \overset{\left(k\right)}{\geq }nh\left(Y|X\right)-h\left(Y_{i}^{n}|X_{i}^{n},J\left(i\right),S\left(i\right),C_{n}\right)-H\left(S\left(i\right)|J,Z^{n},C_{n}\right)\\ \; & \overset{\left(l\right)}{\geq }nh\left(Y|X\right)-h\left(Y_{i}^{n}|X_{i}^{n},J\left(i\right),S\left(i\right),C_{n}\right)-n\delta_{n}^{\prime }\\ \; & \overset{\left(m\right)}{\geq }nh\left(Y|X\right)-n\left(h\left(Y|X,U\right)+\delta_{n}\right)-n\delta_{n}^{\prime }\\ \; & =n(I\left(Y;U|X\right)-\delta_{n}-\delta_{n}^{\prime })\\ \; & =n(h\left(U|X\right)-h\left(U|Y\right)-\delta_{n}-\delta_{n}^{\prime }), \end{array}$$

where

(i) follows as J(i) and S(i) are functions of $Y_{i}^{n}$ for given codebook $C_{n}$,
(j) follows since $\left(Y_{i}^{n},X_{i}^{n}\right)$ are independent of $C_{n}$, and the Markov chain $S\left(i\right)-\left(X_{i}^{n},J\left(i\right)\right)-Z^{n}$ holds,
(k) follows because conditioning reduces entropy and $S\left(i\right)-(J\left(i\right),Z^{n})-J\setminus J\left(i\right)$ is applied,
(l) follows by applying Fano’s inequality since S(i) can be reliably reconstructed from $\left(J,Z^{n}\right)$ for given codebook $C_{n}$, and $\delta_{n}^{\prime }↓0$ as δ↓0 and n→∞,
(m) is due to (A19).

From (A23) and (A24), we have that

(A25) $$\begin{array}{cc} \;\frac{1}{n}I\left(X_{i}^{n};J\left(i\right)|C_{n}\right) & \leq h\left(U|Z\right)-h\left(U|X\right)+R_{I}+6\delta +\delta_{n}+\delta_{n}^{\prime }\\ \; & =I\left(X;U\right)-I\left(Z;U\right)+R_{I}+6\delta +\delta_{n}+\delta_{n}^{\prime }\\ \; & \leq R_{L}+\delta \end{array}$$

for sufficiently large *n*.

Finally, by using the selection lemma [34] (Lemma 2.2), from, e.g., (A16) and (A25), there exists at least one good codebook satisfying all the conditions in Definition 1 for large enough *n*.

### Appendix A.3. Converse Part

We consider a more relaxed case where *W* is uniformly distributed on I, and (13), (15), (17) and (18) in Definition 1 are replaced by

(A26) $$\begin{array}{cc} \mathrm{Pr}\{\left(\hat{W},\hat{S(W)}\right)\neq \left(W,S\left(W\right)\right)\} & \leq \delta , \end{array}$$

(A27) $$\begin{array}{cc} \frac{1}{n}H\left(S\left(W\right)|W\right) & \geq R_{S}-\delta , \end{array}$$

(A28) $$\begin{array}{cc} \frac{1}{n}I\left(S\left(W\right);J\left(W\right)|W\right) & \leq \delta , \end{array}$$

(A29) $$\begin{array}{cc} \frac{1}{n}I\left(X_{W}^{n};J\left(W\right)|W\right) & \leq R_{L}+\delta , \end{array}$$

respectively. Other conditions remain unchanged. We shall show that even for this case the outer bound on $R_{G}$ coincides with its inner bound where there is no assumption of the uniformity of *W*. A similar approach was also taken in [12]. We assume that a rate tuple $\left(R_{I},R_{S},R_{J},R_{L}\right)$ is achievable, implying there exists a pair of encoder and decoder (e,d) such that conditions (14), (16), and (A26)–(A29) are satisfied for any δ>0 and large enough *n*. *Analysis of Identification and Secret-key Rates*: The joint entropy of *W* and S(W) can be developed as

(A30) $$\begin{array}{cc} H(W,S(W)) & =H\left(W,S\left(W\right)|Z^{n},J\right)+I\left(W,S\left(W\right);Z^{n},J\right)\\ \; & \overset{\left(a\right)}{=}H\left(W,S\left(W\right)|\hat{W},\hat{S(W)},Z^{n},J\right)+I\left(W,S\left(W\right);J\right)+I\left(W,S\left(W\right);Z^{n}|J\right)\\ \; & \overset{\left(b\right)}{\leq }H\left(W,S\left(W\right)|\hat{W},\hat{S(W)}\right)+I\left(W,S\left(W\right);J\left(W\right)\right)+I\left(W,S\left(W\right);Z^{n}|J\left(W\right)\right)\\ \; & \overset{\left(c\right)}{\leq }n\delta_{n}+I\left(W;J\left(W\right)\right)+I\left(S\left(W\right);J\left(W\right)|W\right)+I\left(W,S\left(W\right);Z^{n}|J\left(W\right)\right)\\ \; & \overset{\left(d\right)}{\leq }n\left(\delta_{n}+\delta \right)+h\left(Z^{n}|J\left(W\right)\right)-h\left(Z^{n}|J\left(W\right),S\left(W\right)\right)\\ \; & \overset{\left(e\right)}{\leq }n\left(\delta_{n}+\delta \right)+h\left(Z^{n}\right)-h\left(Z^{n}|J\left(W\right),S\left(W\right)\right), \end{array}$$

where

(a) holds since $\left(\hat{W},\hat{S(W)}\right)$ is a function of $\left(Z^{n},J\right)$,
(b) follows because conditioning reduces entropy, and only J(W) is possibly dependent on $Z^{n},S\left(W\right)$,
(c) is due to Fano’s inequality with $\delta_{n}=\frac{1}{n}\left(1+\delta \log M_{I}M_{S}\right)$,
(d) follows since (A28) is applied, and *W* is independent of other RVs,
(e) follows because conditioning reduces entropy.

Due to the uniformity of *W* on I, we have that

(A31) $$\begin{array}{c} H\left(W,S\left(W\right)\right)=H\left(W\right)+H\left(S\left(W\right)|W\right)=\log M_{I}+H\left(S\left(W\right)|W\right). \end{array}$$

From (14), (A27), and (A30), it yields that

(A32) $$\begin{array}{c} R_{I}+R_{S}\leq h\left(Z\right)-\frac{1}{n}h\left(Z^{n}|J\left(W\right),S\left(W\right)\right)+3\delta +\delta_{n}. \end{array}$$

*Analysis of Storage Rate*: From (16),

(A33) $$\begin{array}{cc} \;n(R_{J}+\delta ) & \geq \log M_{J}\geq \max_{w\in I}H\left(J\left(w\right)\right)\geq H\left(J\left(W\right)|W\right)\\ \; & =I\left(Y_{W}^{n};J\left(W\right)|W\right)\overset{\left(f\right)}{=}h\left(Y_{W}^{n}\right)-h\left(Y_{W}^{n}|J\left(W\right),W\right)\\ \; & =h\left(Y_{W}^{n}\right)-h\left(Y_{W}^{n}|J\left(W\right),S\left(W\right),W\right)-I\left(S\left(W\right);Y_{W}^{n}|J\left(W\right),W\right)\\ \; & \overset{\left(g\right)}{=}h\left(Y_{W}^{n}\right)-h\left(Y_{W}^{n}|J\left(W\right),S\left(W\right)\right)-H\left(S\left(W\right)|J\left(W\right),W\right)\\ \; & \geq h\left(Y_{W}^{n}\right)-h\left(Y_{W}^{n}|J\left(W\right),S\left(W\right)\right)-H\left(S\left(W\right)|W\right)\\ \; & \overset{\left(h\right)}{\geq }h\left(Z^{n}|J\left(W\right),S\left(W\right)\right)-h\left(Y_{W}^{n}|J\left(W\right),S\left(W\right)\right)+n\left(R_{I}-\left(\delta_{n}+2\delta \right)\right), \end{array}$$

where

(f) holds as *W* is independent of $Y_{W}^{n}$,
(g) holds as *W* is independent of other RVs and S(W) is a function of $Y_{W}^{n}$,
(h) follows because $h\left(Y_{W}^{n}\right)=h\left(Z^{n}\right)=\frac{n}{2}\log\left(2\pi e\right)$, and by combining (A30) and (A31), we obtain that $H\left(S\left(W\right)|W\right)\leq h\left(Z^{n}\right)-h\left(Z^{n}|J\left(W\right),S\left(W\right)\right)-n\left(R_{I}-\left(\delta_{n}+2\delta \right)\right)$.

*Analysis of Privacy-Leakage Rate*: From (A29),

(A34) $$\begin{array}{cc} n(R_{L}+\delta ) & \geq I\left(X_{W}^{n};J\left(W\right)|W\right)\overset{\left(i\right)}{=}h\left(X_{W}^{n}\right)-h\left(X_{W}^{n}|J\left(W\right),W\right)\\ \; & =h\left(X_{W}^{n}\right)-h\left(X_{W}^{n}|J\left(W\right),S\left(W\right),W\right)-I\left(S\left(W\right);X_{W}^{n}|J\left(W\right),W\right)\\ \; & \geq h\left(X_{W}^{n}\right)-h\left(X_{W}^{n}|J\left(W\right),S\left(W\right)\right)-H\left(S\left(W\right)|J\left(W\right),W\right)\\ \; & \geq h\left(X_{W}^{n}\right)-h\left(X_{W}^{n}|J\left(W\right),S\left(W\right)\right)-H\left(S\left(W\right)|W\right)\\ \; & \overset{\left(j\right)}{\geq }h\left(Z^{n}|J\left(W\right),S\left(W\right)\right)-h\left(X_{W}^{n}|J\left(W\right),S\left(W\right)\right)+n\left(R_{I}-\left(\delta_{n}+2\delta \right)\right), \end{array}$$

where

(i) holds as *W* is independent of $X_{W}^{n}$,
(j) follows because $h\left(X_{W}^{n}\right)=h\left(Z^{n}\right)$, and the same reason of (h) in (A33) is used.

For further evaluations of (A32)–(A34), we scrutinize a lower bound on $h(Z^{n}|J\left(W\right),S\left(W\right))$ and an upper bound on $h(Y_{W}^{n}|J\left(W\right),S\left(W\right))$ with fixed $h(X_{W}^{n}|J\left(W\right),S\left(W\right))$ by applying the conditional EPI [27] (Lemma II). It is a key to set

(A35) $$\begin{array}{c} \;\frac{1}{n}h\left(X_{W}^{n}|J\left(W\right),S\left(W\right)\right)=\frac{1}{2}\log\left(2\pi e(\alpha \rho_{1}^{2}+1-\rho_{1}^{2})\right) \end{array}$$

with some 0<α≤1. Indeed, this is a reasonable setting because $\frac{1}{2}\log\left(2\pi e\right)\geq \frac{1}{n}h\left(X_{W}^{n}|J\left(W\right),S\left(W\right)\right)\geq \frac{1}{2}\log\left(2\pi e\left(1-\rho_{1}^{2}\right)\right)$. The lower bound is obtained from $\frac{1}{n}h\left(X_{W}^{n}|J\left(W\right),S\left(W\right)\right)\geq \frac{1}{n}h\left(X_{W}^{n}|Y_{W}^{n},J\left(W\right),S\left(W\right)\right)=\frac{1}{n}h\left(X_{W}^{n}|Y_{W}^{n}\right)$ due to the fact that (J(W),S(W)) is a function of $Y_{W}^{n}$. For α=0, it is not possible to achieve such point, and the reason will be explained right after Equation (A40).

In the direction from *X* to *Z*, by applying the conditional EPI [27] (Lemma II) to the first equality in (10), it follows that

(A36) $$\begin{array}{cc} e^{\frac{2}{n}h\left(Z^{n}|J\left(W\right),S\left(W\right)\right)} & \geq e^{\frac{2}{n}h\left(\rho_{2}X_{W}^{n}|J\left(W\right),S\left(W\right)\right)}+e^{\frac{2}{n}h\left(N_{2}^{n}|J\left(W\right),S\left(W\right)\right)}\\ \; & \overset{\left(k\right)}{=}\rho_{2}^{2}e^{\frac{2}{n}h\left(X_{W}^{n}|J\left(W\right),S\left(W\right)\right)}+e^{\frac{2}{n}h\left(N_{2}^{n}\right)}\\ \; & =\rho_{2}^{2}\left(2\pi e(\alpha \rho_{1}^{2}+1-\rho_{1}^{2})\right)+2\pi e\left(1-\rho_{2}^{2}\right)\\ \; & =2\pi e(\alpha \rho_{1}^{2}\rho_{2}^{2}+1-\rho_{1}^{2}\rho_{2}^{2}), \end{array}$$

where (k) holds as $N_{2}^{n}$ is independent of (J(W),S(W)), and as a deduction,

(A37) $$\begin{array}{c} \;\frac{1}{n}h\left(Z^{n}|J\left(W\right),S\left(W\right)\right)\geq \frac{1}{2}\log\left(2\pi e\left(\alpha \rho_{1}^{2}\rho_{2}^{2}+1-\rho_{1}^{2}\rho_{2}^{2}\right)\right). \end{array}$$

In the opposite direction (from *X* to *Y*), again applying the conditional EPI [27] (Lemma II) to (9), we have that

(A38) $$\begin{array}{cc} e^{\frac{2}{n}h\left(X_{W}^{n}|J\left(W\right),S\left(W\right)\right)} & \geq e^{\frac{2}{n}h\left(\rho_{1}Y_{W}^{n}|J\left(W\right),S\left(W\right)\right)}+e^{\frac{2}{n}h\left({\left(N_{1}^{\prime }\right)}^{n}\right)}, \end{array}$$

where the inequality holds as ${\left(N_{1}^{\prime }\right)}^{n}$ is also independent of (J(W),S(W)), meaning that

(A39) $$\begin{array}{cc} 2\pi e(\alpha \rho_{1}^{2}+1-\rho_{1}^{2}) & \geq \rho_{1}^{2}e^{\frac{2}{n}h\left(Y_{W}^{n}|J\left(W\right),S\left(W\right)\right)}+2\pi e\left(1-\rho_{1}^{2}\right) \end{array}$$

and thus

(A40) $$\begin{array}{cc} e^{\frac{2}{n}h\left(Y_{W}^{n}|J\left(W\right),S\left(W\right)\right)} & \leq 2\pi e\alpha ,\\ \frac{1}{n}h\left(Y_{W}^{n}|J\left(W\right),S\left(W\right)\right) & \leq \frac{1}{2}\log\left(2\pi e\alpha \right), \end{array}$$

which is not derivable from the first equation in (1) of the original system. As previously mentioned for the case in which α=0, in (A40), since RV *Y* has unit variance, it is required that the joint entropy H(J(W),S(W)) should be infinity, but this value is impossible to achieve for finite sets S and J.

Now plugging (A35), (A37), and (A40) into (A32)–(A34), we obtain that

(A41) $$\begin{array}{cc} \; & R_{I}+R_{S}\leq \frac{1}{2}\log\left(\frac{1}{\alpha \rho_{1}^{2}\rho_{2}^{2}+1-\rho_{1}^{2}\rho_{2}^{2}}\right)+3\delta +\delta_{n}, \end{array}$$

(A42) $$\begin{array}{cc} \; & R_{J}\geq \frac{1}{2}\log\left(\frac{\alpha \rho_{1}^{2}\rho_{2}^{2}+1-\rho_{1}^{2}\rho_{2}^{2}}{\alpha }\right)+R_{I}-\left(3\delta +\delta_{n}\right), \end{array}$$

(A43) $$\begin{array}{cc} \; & R_{L}\geq \frac{1}{2}\log\left(\frac{\alpha \rho_{1}^{2}\rho_{2}^{2}+1-\rho_{1}^{2}\rho_{2}^{2}}{\alpha \rho_{1}^{2}+1-\rho_{1}^{2}}\right)+R_{I}-\left(3\delta +\delta_{n}\right). \end{array}$$

Eventually, by letting n→∞ and δ↓0, from (A41)–(A43), we can see that the capacity region is contained in the right-hand side of (19).

## Appendix B. Proof Sketch of Equation (20)

In this section, we highlight the proof of the chosen-secret BIS model. The parts that follow directly from the same arguments in Appendix A are omitted.

### Appendix B.1. Achievability Part

The proof slightly differs from the one in Appendix A, in which the encoder and decoder of the generated-secret BIS model are used as components inside the encoder and decoder of the chosen-secret BIS model as shown in Figure A2. In order to avoid confusion in the subsequent arguments, we define some new notation used only in this part. The pairs $\left(J_{C}\left(i\right),S_{C}\left(i\right)\right)$ and $\left(J_{G}\left(i\right),S_{G}\left(i\right)\right)$ denote the helper and secret key for individual *i* generated by the encoders of chosen- and generated-secret BIS models, respectively. To encode $Y_{i}^{n}$ for i∈I, the component inside the encoder first generates $\left(J_{G}\left(i\right),S_{G}\left(i\right)\right)$ and then uses $S_{G}\left(i\right)$ to mask $S_{C}\left(i\right)$ by executing one-time pad operation $S_{C}\left(i\right)⊕S_{G}\left(i\right)$, where ⊕ denotes the addition modulo $M_{S}$. The helper data $J_{C}\left(i\right)$ is the combined information of $J_{G}\left(i\right)$ and the masked data, i.e.,

(A44) $$\begin{array}{c} J_{C}\left(i\right)=\left(J_{G}\left(i\right),S_{C}\left(i\right)⊕S_{G}\left(i\right)\right). \end{array}$$

For decoding the identified user W=i, it first uses the component decoder to estimate ($\hat{W},\hat{S_{G}\left(W\right)}$) and then the secret key is retrieved from

(A45) $$\begin{array}{c} \hat{S_{C}\left(W\right)}=S_{C}\left(\hat{W}\right)⊕S_{G}\left(\hat{W}\right)⊖\hat{S_{G}\left(W\right)}, \end{array}$$

where ⊖ denotes the subtraction modulo $M_{S}$. This technique is also used in [8,11,13]. *Analysis of Error Probability*: For individual W=i, the operation at the decoder (A45) means that $\left(\hat{W},\hat{S_{C}\left(W\right)}\right)=\left(W,S_{C}\left(W\right)\right)$ if and only if $\left(\hat{W},\hat{S_{G}\left(W\right)}\right)=\left(W,S_{G}\left(W\right)\right)$. In (A16), it is revealed that $\mathrm{Pr}\{\left(\hat{W},\hat{S_{G}\left(W\right)}\right)\neq \left(W,S_{G}\left(W\right)\right)|W=i\}\leq 4\delta .$ Therefore, the error probability of the chosen-secret BIS model can also be bounded by

(A46) $$\begin{array}{c} \mathrm{Pr}\{\left(\hat{W},\hat{S_{C}\left(W\right)}\right)\neq \left(W,S_{C}\left(W\right)\right)|W=i\}\leq 4\delta \end{array}$$

for large enough *n*.

*Analyses of Identification and Secret-key Rates*: Equations (14) and (15) are straightforward from the parameter settings.

*Analysis of Storage Rate*: From (A44), the total storage rate is bounded by

(A47) $$\begin{array}{cc} \underset{\mathrm{the}\;\mathrm{rate}\;\mathrm{of}\;J_{G}\left(i\right)}{\underset{︸}{I\left(Y;U\right)-I\left(Z;U\right)+R_{I}+6\delta }}+\underset{\mathrm{the}\;\mathrm{rate}\;\mathrm{of}\;S_{C}\left(i\right)⊕S_{G}\left(i\right)}{\underset{︸}{I\left(Z;U\right)-R_{I}-2\delta }}\; & =I(Y;U)+4\delta \\ \; & =\frac{1}{2}\log\left(\frac{1}{\alpha }\right)+4\delta . \end{array}$$

*Analysis of Secrecy-Leakage Rate*: From the similar argument of [11] (Equation (48)), it was shown that

(A48) $$\begin{array}{cc} I & \left(J_{C}\left(i\right);S_{C}\left(i\right)|C_{n}\right)\leq I\left(J_{G}\left(i\right);S_{G}\left(i\right)|C_{n}\right)+\log M_{S}-H\left(S_{G}\left(i\right)|C_{n}\right), \end{array}$$

and by substituting (A22) into (A48), the secrecy-leakage rate of the chosen-secret BIS model is bounded by

(A49) $$\begin{array}{c} \frac{1}{n}I\left(J_{C}\left(i\right);S_{C}\left(i\right)|C_{n}\right)\leq 10\delta \end{array}$$

for large enough *n*.

*Analysis of Privacy-Leakage Rate*: It can be proved that

(A50) $$\begin{array}{c} I\left(X_{i}^{n};J_{C}\left(i\right)|C_{n}\right)=I\left(X_{i}^{n};J_{G}\left(i\right)|C_{n}\right). \end{array}$$

To verify this, first one can easily see that

(A51) $$\begin{array}{c} I\left(X_{i}^{n};J_{C}\left(i\right)|C_{n}\right)=I\left(X_{i}^{n};J_{G}\left(i\right),S_{C}\left(i\right)⊕S_{G}\left(i\right)|C_{n}\right)\geq I\left(X_{i}^{n};J_{G}\left(i\right)|C_{n}\right). \end{array}$$

Meanwhile, by the same analogy to the development of ([11] Equation (49)), it also holds that

(A52) $$\begin{array}{c} I\left(X_{i}^{n};J_{C}\left(i\right)|C_{n}\right)\leq I\left(X_{i}^{n};J_{G}\left(i\right)|C_{n}\right). \end{array}$$

From (A51) and (A52), (A50) clearly holds. By invoking the result of (A25), from (A50), the privacy-leakage rate can also be made that

(A53) $$\begin{array}{c} \frac{1}{n}I\left(X_{i}^{n};J_{C}\left(i\right)|C_{n}\right)\leq R_{L}+\delta \end{array}$$

for large enough *n*.

Finally, by using the selection lemma [34] (Lemma 2.2), there is at least one good codebook satisfying all the conditions in Definition 2 for large enough *n*.

### Appendix B.2. Converse Part

As seen in the converse proof of the generated-secret BIS model, we also consider the case in which *W* is uniformly distributed on I. Suppose that a pair $\left(R_{I},R_{S},R_{J},R_{L}\right)$ is achievable and fix α such that the condition in (A35) is satisfied.

For the analyses of identification, secret-key, and privacy-leakage rates, the reader should refer to the discussions around (A32) and (A34). We argue only the bound on $R_{J}$, which is different from the one seen in the generated-secret BIS model. *Analysis of Storage Rate*:

(A54) $$\begin{array}{cc} n(R_{J}+\delta ) & \geq \log M_{J}\geq \max_{w\in I}H\left(w\right)\geq H\left(J\left(W\right)|W\right)\\ & \overset{\left(a\right)}{=}I\left(Y_{W}^{n},S\left(W\right);J\left(W\right)|W\right)=I\left(S\left(W\right);J\left(W\right)|W\right)+I\left(Y_{W}^{n};J\left(W\right)|W,S\left(W\right)\right)\\ \; & \geq I\left(Y_{W}^{n};J\left(W\right)|W,S\left(W\right)\right)\overset{\left(b\right)}{=}h\left(Y_{W}^{n}\right)-h\left(Y_{W}^{n}|J\left(W\right),S\left(W\right)\right)\\ \; & \overset{\left(c\right)}{\geq }\frac{n}{2}\log\left(2\pi e\right)-\frac{n}{2}\log\left(2\pi e\alpha \right)=\frac{n}{2}\log\left(\frac{1}{\alpha }\right), \end{array}$$

where

(a) follows since J(W) is a function of $\left(Y_{W}^{n},S\left(W\right)\right)$,
(b) follows as *W* is independent of other RVs and S(W) is chosen independently of $Y_{W}^{n}$,
(c) follows because (A40) is applied.

Then, we have that

(A55) $$\begin{array}{c} R_{J}\geq \frac{1}{2}\log\left(\frac{1}{\alpha }\right)-\delta . \end{array}$$

By letting n→∞ and δ↓0, the capacity region of the chosen-secret BIS model is contained in the right-hand side of (20).

## Appendix C. Convexity of the Regions $R_{G}$ and $R_{C}$

Here, we only verify that the region $R_{G}$ is convex as the proof for $R_{C}$ follows similarly. We begin with the case in which $\rho_{1}^{2}\rho_{2}^{2}>0$. We first define $\eta =\frac{1}{\alpha \rho_{1}^{2}\rho_{2}^{2}+1-\rho_{1}^{2}\rho_{2}^{2}},$ and then it follows that $\alpha =\frac{1}{\rho_{1}^{2}\rho_{2}^{2}}\left(\frac{1}{\eta }-\left(1-\rho_{1}^{2}\rho_{2}^{2}\right)\right).$ Therefore, the constraints of $R_{J}$ and $R_{L}$ in (19) can be transformed as follows:

(A56) $$\begin{array}{cc} R_{J} & \geq \frac{1}{2}\log\left(\frac{\frac{1}{\rho_{1}^{2}\rho_{2}^{2}}\left(\frac{1}{\eta }-\left(1-\rho_{1}^{2}\rho_{2}^{2}\right)\right)\rho_{1}^{2}\rho_{2}^{2}+1-\rho_{1}^{2}\rho_{2}^{2}}{\frac{1}{\rho_{1}^{2}\rho_{2}^{2}}\left(\frac{1}{\eta }-\left(1-\rho_{1}^{2}\rho_{2}^{2}\right)\right)}\right)+R_{I}\\ \; & =\frac{1}{2}\log\left(\frac{\rho_{1}^{2}\rho_{2}^{2}}{1-(1-\rho_{1}^{2}\rho_{2}^{2})\eta }\right)+R_{I}\\ \; & =-\frac{1}{2}\log\left(1-(1-\rho_{1}^{2}\rho_{2}^{2})\eta \right)+\log\left|\rho_{1}\rho_{2}\right|+R_{I}, \end{array}$$

and

(A57) $$\begin{array}{cc} R_{L} & \geq \frac{1}{2}\log\left(\frac{\frac{1}{\rho_{1}^{2}\rho_{2}^{2}}\left(\frac{1}{\eta }-\left(1-\rho_{1}^{2}\rho_{2}^{2}\right)\right)\rho_{1}^{2}\rho_{2}^{2}+1-\rho_{1}^{2}\rho_{2}^{2}}{\frac{1}{\rho_{1}^{2}\rho_{2}^{2}}\left(\frac{1}{\eta }-\left(1-\rho_{1}^{2}\rho_{2}^{2}\right)\right)\rho_{1}^{2}+1-\rho_{1}^{2}}\right)+R_{I}\\ \; & =\frac{1}{2}\log\left(\frac{\rho_{2}^{2}}{1-(1-\rho_{2}^{2})\eta }\right)+R_{I}\\ \; & =-\frac{1}{2}\log\left(1-(1-\rho_{2}^{2})\eta \right)+\log\left|\rho_{2}\right|+R_{I}. \end{array}$$

Since $0<|\rho_{1}\left|,\right|\rho_{2}|<1$, and 0<α≤1, it holds that $\frac{1-\rho_{2}^{2}}{\alpha \rho_{1}^{2}\rho_{2}^{2}+1-\rho_{1}^{2}\rho_{2}^{2}}<\frac{1-\rho_{1}^{2}\rho_{2}^{2}}{\alpha \rho_{1}^{2}\rho_{2}^{2}+1-\rho_{1}^{2}\rho_{2}^{2}}<1$, indicating the values of $1-(1-\rho_{1}^{2}\rho_{2}^{2})\eta$ and $1-(1-\rho_{2}^{2})\eta$ are positive. Now the region in (19) can also be expressed as follows:

(A58) $$\begin{array}{cc} R_{G}=\{\left(R_{I},R_{S},R_{J},R_{L}\right):\;\; & R_{I}+R_{S}\leq \frac{1}{2}\log\eta ,\\ \; & R_{J}\geq -\frac{1}{2}\log\left(1-(1-\rho_{1}^{2}\rho_{2}^{2})\eta \right)+\log\left|\rho_{1}\rho_{2}\right|+R_{I},\\ \; & R_{L}\geq -\frac{1}{2}\log\left(1-(1-\rho_{2}^{2})\eta \right)+\log\left|\rho_{2}\right|+R_{I},\\ \; & R_{I}\geq 0,\;\;R_{S}\geq 0\;\mathrm{for}\;\mathrm{some}\;1\leq \eta <\frac{1}{1-\rho_{1}^{2}\rho_{2}^{2}}\}. \end{array}$$

Suppose that the rate tuples $R^{1}=\left(R_{I}^{1},R_{S}^{1},R_{J}^{1},R_{L}^{1}\right)$ and $R^{2}=\left(R_{I}^{2},R_{S}^{2},R_{J}^{2},R_{L}^{2}\right)$ are achievable tuples for $\eta_{1}$ and $\eta_{2}$, respectively. Without loss of generality, we assume that $1\leq \eta_{1}\leq \eta_{2}<\frac{1}{1-\rho_{1}^{2}\rho_{2}^{2}}$. Next, let us consider linear combinations of these tuples. For 0≤λ≤1, we have that

(A59) $$\begin{array}{cc} \lambda \left(R_{I}^{1}+R_{S}^{1}\right)+\left(1-\lambda \right)\left(R_{I}^{2}+R_{S}^{2}\right) & \leq \frac{1}{2}\left(\lambda \log\eta_{1}+\left(1-\lambda \right)\log\eta_{2}\right)\\ \; & \overset{\left(a\right)}{\leq }\frac{1}{2}\log\left(\lambda \eta_{1}+\left(1-\lambda \right)\eta_{2}\right)\\ \; & \overset{\left(b\right)}{=}\frac{1}{2}\log\eta^{\prime }, \end{array}$$

where

(a) follows as logx(x>0) is a concave function,
(b) holds as we define $\eta^{\prime }=\lambda \eta_{1}+\left(1-\lambda \right)\eta_{2}$.

Now take a look into the bound on the storage rate

(A60) $$\begin{array}{cc} \lambda R_{J}^{1}+\left(1-\lambda \right)R_{J}^{2} & \geq -\lambda \frac{1}{2}\log\left(1-\left(1-\rho_{1}^{2}\rho_{2}^{2}\right)\eta_{1}\right)-\left(1-\lambda \right)\frac{1}{2}\log\left(1-\left(1-\rho_{1}^{2}\rho_{2}^{2}\right)\eta_{2}\right)\\ \; & \;\;\;\;\;+\log|\rho_{1}\rho_{2}|+R_{I}\\ \; & \overset{\left(c\right)}{\geq }-\frac{1}{2}\log\left(1-\left(1-\rho_{1}^{2}\rho_{2}^{2}\right)\left(\lambda \eta_{1}+\left(1-\lambda \right)\eta_{2}\right)\right)+\log\left|\rho_{1}\rho_{2}\right|+R_{I}\\ \; & =-\frac{1}{2}\log\left(1-\left(1-\rho_{1}^{2}\rho_{2}^{2}\right)\eta^{\prime }\right)+\log\left|\rho_{1}\rho_{2}\right|+R_{I}, \end{array}$$

where (c) follows because f(x)=−log(1−x)(x<1) is a convex function. Likewise, we can also show that

(A61) $$\begin{array}{c} \lambda R_{L}^{1}+\left(1-\lambda \right)R_{L}^{2}\geq -\frac{1}{2}\log\left(1-\left(1-\rho_{2}^{2}\right)\eta^{\prime }\right)+\log\left|\rho_{2}\right|+R_{I}. \end{array}$$

From (A59)–(A61), we see that there exists an $\eta^{\prime }$, where $\eta_{1}\leq \eta^{\prime }\leq \eta_{2}$, that satisfies $\lambda R^{1}+\left(1-\lambda \right)R^{2}\in R_{G}$.

For the other case (when $\rho_{1}^{2}\rho_{2}^{2}=0$), the right-hand sides of each constraint in $R_{G}$ become simpler forms, and it is easier to check that both regions are convex by applying Jensen’s inequality to the logarithmic function. Accordingly, this concludes that the region $R_{G}$ is convex.
